# Supplementary material for: A method for an unbiased estimate of cross-ancestry genetic correlation using individual-level data
Source: Nat Commun. 2023 Feb 9;14:722. doi: 10.1038/s41467-023-36281-x (PMC9911789; doi:10.1038/s41467-023-36281-x)
Supplement: Supplementary file 1 — Supplementary Information [file 41467_2023_36281_MOESM1_ESM.pdf]

Supplementary information

Supplementary Table 1: Theoretical bias factors and estimated genomic relationships when using the standard scaling (α = -0.5).

Number of random population samples (n)	Bias factor	Mean (±SD) of off-diagonals in estimated GRM		Mean (±SD) of diagonals in estimated GRM	
		Existing method (equation 5)	Proposed method (equation 2)	Existing method (equation 5)	Proposed method (equation 2)
10	-0.1	-0.0961±0.0035	-0.0094±0.0044	0.8592±0.0032	1.0±0.0037
100	-0.01	-0.0101±0.0045	0.0000±0.0044	0.9936±0.0127	1.0±0.0112
1000	-0.001	-0.0010±0.0046	0.0000±0.0046	0.9997±0.0143	1.0±0.0138
10000	-0.0001	-0.0001±0.0046	0.0000±0.0046	1.0003±0.0135	1.0±0.0132

GRM was estimated for White British based on 500,000 SNPs with various sample sizes. Diagonals and off-diagonals are biasedly estimated when using existing method (i.e. equation 5), noting that the bias factor is $f_{bias} = -1/n * var(x) * [2p_l(1-p_l)]^{2\alpha} = -1/n$ (with α = -0.50) (see equations 5-8 in Methods). When correcting the bias factor, the proposed method (i.e., equation 2 or 8) provides unbiased estimates.

Supplementary Table 2: Slightly biased estimates of SNP-based heritability when using the expected variance in scaling genotypic coefficients in constructing GRM.

	Using actual variance (var(x)) in scaling genotypic coefficients to estimate GRM		Using expected variance (2p(1-p)) in scaling genotypic coefficients to estimate GRM	
**Ancestry**	**Estimated h^2** (±SE)	**P-value**	**Estimated h^2** (±SE)	**P-value**
British	0.479±0.013	0.1062	0.484±0.013	0.2184
South Asian	0.483±0.013	0.1910	0.474±0.013	0.0455
Africa	0.508±0.012	0.5050	0.465±0.012	0.0035
Mixed ancestry	0.489±0.009	0.2216	0.476±0.010	0.0163

The true heritability was 0.50. Mean estimate and SE were calculated over 500 replicates. P-values were based on Wald's test statistics for testing the null hypothesis of estimated h^2= 0.50 (i.e., a two-sided test). In simulation, the causal SNP effects were scaled by actual variance (see method section for details). We observed biased heritability estimates (red coloured) in South Asian, African and mixed ancestries when the estimation was based on the expected variance instead of the actual variance.

Supplementary Table 3: Inferring scaling factors in White British ancestry cohort for LDAK-thin-α and GCTA-α models.

Log-likelihood of White British ancestry cohort (n=30000), LDAK-thin-α model							
**Traits**	**α= -0.625**	**α= -0.50**	**α= -0.375**	**α= -0.25**	**α= -0.125**	**α =0**	**α =0.125**
**BMI**	-14648.386	-14641.042	-14638.387	-14638.168	-14639.145	-14640.688	-14642.484
**Standing Height**	-13981.958	-13939.456	-13919.496	-13912.319	-13912.013	-13915.356	-13920.646
**Waist circumference**	-14701.715	-14697.099	-14695.797	-14696.171	-14697.357	-14698.923	-14700.655
**Hip circumference**	-14681.115	-14674.272	-14671.691	-14671.314	-14672.012	-14673.214	-14674.641
**Waist-hip ratio**	-14746.071	-14741.714	-14739.861	-14739.388	-14739.669	-14740.370	-14741.302
**Weight**	-14587.158	-14578.471	-14575.913	-14576.464	-14578.471	-14581.111	-14583.984
**Multivariate model[a]**	**-87346.403**	**-87272.054**	**-87241.145**	**-87233.824**	**-87238.667**	**-87249.662**	**-87263.712**
**AIC[b]**	**174704.806**	**174556.108**	**174494.29**	**174479.648**	**174489.334**	**174511.324**	**174539.424**
**ΔAIC[c]**	**225.158**	**76.46**	**14.642**	**0**	**9.686**	**31.676**	**59.776**

Log-likelihood of White British ancestry cohort (n=30000), GCTA-α model							
**Traits**	**α= -0.625**	**α= -0.50**	**α= -0.375**	**α= -0.25**	**α= -0.125**	**α =0**	**α =0.125**
**BMI**	-14646.809	-14637.704	-14633.573	-14632.214	-14632.345	-14633.272	-14634.626
**Standing Height**	-13916.334	-13869.658	-13846.069	-13836.442	-13834.900	-13838.006	-13843.798
**Waist circumference**	-14703.271	-14697.264	-14694.827	-14694.348	-14694.914	-14696.035	-14697.451
**Hip circumference**	-14680.958	-14673.030	-14669.419	-14668.202	-14668.272	-14669.032	-14670.163
**Waist-hip ratio**	-14744.532	-14739.444	-14737.009	-14736.091	-14736.032	-14736.471	-14737.202
**Weight**	-14579.028	-14568.363	-14564.171	-14563.515	-14564.692	-14566.794	-14569.344
**Multivariate model[a]**	**-87270.932**	**-87185.463**	**-87145.068**	**-87130.812**	**-87131.155**	**-87139.61**	**-87152.584**
**AIC[b]**	**174553.864**	**174382.926**	**174302.136**	**174273.624**	**174274.31**	**174291.22**	**174317.168**
**ΔAIC[c]**	**280.24**	**109.302**	**28.512**	**0**	**0.686**	**17.596**	**43.544**

[a]Multivariate linear mixed model was used to get the log-likelihood of the scaling factor where residual and genetic correlations between traits were fixed as zero, i.e. the log-likelihood of this multivariate linear mixed model is the sum of log-likelihood values from the trait-specific analyses. [b]Akaike Information Criterion (AIC) $= 2k - 2ln(L)$ where $2ln(L)$ is the logarithm of the maximum likelihood from the model and k is the number of model parameters in the model. [c]ΔAIC = AIC – AIC of the best model with the optimal α. The best model is red highlighted.

Supplementary Table 4: Inferring scaling factors in Other European ancestry cohort for LDAK-thin-α and GCTA-α models.

Log-likelihood of Other European ancestry cohort (n=26457), LDAK-thin-α model							
**Traits**	**α= -0.625**	**α= -0.50**	**α= -0.375**	**α= -0.25**	**α= -0.125**	**α =0**	**α =0.125**
**BMI**	-12825.531	-12817.869	-12814.507	-12813.513	-12813.782	-12814.721	-12816.014
**Standing Height**	-12314.739	-12288.168	-12277.591	-12275.302	-12277.133	-12281.057	-12286.098
**Waist circumference**	-12894.232	-12889.131	-12886.667	-12885.639	-12885.384	-12885.563	-12885.998
**Hip circumference**	-12875.624	-12868.142	-12864.204	-12862.319	-12861.599	-12861.555	-12861.916
**Waist-hip ratio**	-12918.195	-12914.027	-12911.747	-12910.574	-12910.057	-12909.947	-12910.099
**Weight**	-12816.469	-12806.169	-12800.980	-12798.712	-12798.070	-12798.346	-12799.151
**Multivariate model[a]**	**-76644.790**	**-76583.506**	**-76555.696**	**-76546.059**	**-76546.025**	**-76551.189**	**-76559.276**
**AIC[b]**	**153301.58**	**153179.012**	**153123.392**	**153104.118**	**153104.05**	**153114.378**	**153130.552**
**ΔAIC[c]**	**197.53**	**74.962**	**19.342**	**0.068**	**0**	**10.328**	**26.502**

Log-likelihood of Other European ancestry cohort (n=26457), GCTA-α model							
**Traits**	**α= -0.625**	**α= -0.50**	**α= -0.375**	**α= -0.25**	**α= -0.125**	**α =0**	**α =0.125**
**BMI**	-12822.089	-12814.182	-12810.608	-12809.513	-12809.781	-12810.791	-12812.197
**Standing Height**	-12282.774	-12255.861	-12243.939	-12240.204	-12240.845	-12243.901	-12248.364
**Waist circumference**	-12889.703	-12884.144	-12881.440	-12880.387	-12880.267	-12880.679	-12881.398
**Hip circumference**	-12870.057	-12862.691	-12858.841	-12857.059	-12856.466	-12856.567	-12857.083
**Waist-hip ratio**	-12916.542	-12911.875	-12909.306	-12908.041	-12907.572	-12907.594	-12907.922
**Weight**	-12809.056	-12798.907	-12793.705	-12791.405	-12790.771	-12791.108	-12792.022
**Multivariate model[a]**	**-76590.221**	**-76527.660**	**-76497.839**	**-76486.609**	**-76485.702**	**-76490.640**	**-76498.986**
**AIC[b]**	**153192.442**	**153067.32**	**153007.678**	**152985.218**	**152983.404**	**152993.28**	**153009.972**
**ΔAIC[c]**	**209.038**	**83.916**	**24.274**	**1.814**	**0**	**9.876**	**26.568**

[a]Multivariate linear mixed model was used to get the log-likelihood of the scaling factor where residual and genetic correlations between traits were fixed as zero, i.e. the log-likelihood of this multivariate linear mixed model is the sum of log-likelihood values from the trait-specific analyses. [b]Akaike Information Criterion (AIC) $= 2k - 2ln(L)$ where $2ln(L)$ is the logarithm of the maximum likelihood from the model and k is the number of model parameters in the model. [c]ΔAIC = AIC – AIC of the best model with the optimal α. The best model is red highlighted.

Supplementary Table 5: Inferring scaling factors in South Asian ancestry cohort for LDAK-thin-α and GCTA-α models

Log-likelihood of South Asian ancestry cohort (n=6199), LDAK-thin-α model

**Traits**	**α= -1**	**α= -0.875**	**α= -0.75**	**α= -0.625**	**α= -0.50**	**α= -0.375**	**α= -0.25**	**α= -0.125**	**α= 0**	**α= 0.125**
**BMI**	-2850.005	-2848.154	-2847.217	-2847.138	-2847.531	-2848.068	-2848.596	-2849.070	-2849.483	-2849.846
**Standing Height**	-2841.233	-2832.923	-2826.611	-2823.219	-2822.141	-2822.336	-2823.049	-2823.885	-2824.672	-2825.355
**Waist circumference**	-2902.485	-2901.083	-2900.065	-2899.583	-2899.510	-2899.643	-2899.849	-2900.068	-2900.271	-2900.453
**Hip circumference**	-2900.520	-2900.467	-2900.895	-2901.583	-2902.269	-2902.831	-2903.255	-2903.571	-2903.809	-2903.994
**Waist-hip ratio**	-2907.148	-2906.052	-2904.386	-2902.869	-2901.899	-2901.465	-2901.402	-2901.555	-2901.812	-2902.113
**Weight**	-2892.291	-2889.713	-2887.985	-2887.286	-2887.301	-2887.651	-2888.106	-2888.568	-2888.999	-2889.392
**Multivariate model[a]**	**-17293.682**	**-17278.392**	**-17267.159**	**-17261.678**	**-17260.651**	**-17261.994**	**-17264.257**	**-17266.717**	**-17269.046**	**-17271.253**
**AIC[b]**	**34599.364**	**34568.784**	**34546.318**	**34535.356**	**34533.302**	**34535.988**	**34540.514**	**34545.434**	**34550.092**	**34554.506**
**ΔAIC[c]**	**66.062**	**35.482**	**13.016**	**2.054**	**0**	**2.686**	**7.212**	**12.132**	**16.79**	**21.204**

Log-likelihood of South Asian ancestry cohort (n=6199), GCTA-α model

**Traits**	**α= -1**	**α= -0.875**	**α= -0.75**	**α= -0.625**	**α= -0.50**	**α= -0.375**	**α= -0.25**	**α= -0.125**	**α= 0**	**α= 0.125**
**BMI**	-2848.455	-2847.351	-2847.214	-2847.781	-2848.609	-2849.413	-2850.099	-2850.661	-2851.126	-2851.517
**Standing Height**	-2836.662	-2828.602	-2823.144	-2820.685	-2820.319	-2820.979	-2821.974	-2822.979	-2823.877	-2824.636
**Waist circumference**	-2901.579	-2900.588	-2900.065	-2899.999	-2900.194	-2900.474	-2900.752	-2900.999	-2901.212	-2901.394
**Hip circumference**	-2898.808	-2899.118	-2900.006	-2901.114	-2902.113	-2902.885	-2903.446	-2903.850	-2904.145	-2904.366
**Waist-hip ratio**	-2907.145	-2906.279	-2905.106	-2904.144	-2903.606	-2903.439	-2903.509	-2903.706	-2903.956	-2904.219
**Weight**	-2889.802	-2887.788	-2886.776	-2886.719	-2887.201	-2887.849	-2888.489	-2889.059	-2889.557	-2889.993
**Multivariate model[a]**	**-17282.451**	**-17269.726**	**-17262.311**	**-17260.442**	**-17262.042**	**-17265.039**	**-17268.269**	**-17271.254**	**-17273.873**	**-17276.125**
**AIC[b]**	**34576.902**	**34551.452**	**34536.622**	**34532.884**	**34536.084**	**34542.078**	**34548.538**	**34554.508**	**34559.746**	**34564.25**
**ΔAIC[c]**	**44.018**	**18.568**	**3.738**	**0**	**3.2**	**9.194**	**15.654**	**21.624**	**26.862**	**31.366**

[a]Multivariate linear mixed model was used to get the log-likelihood of the scaling factor where residual and genetic correlations between traits were fixed as zero, i.e. the log-likelihood of this multivariate linear mixed model is the sum of log-likelihood values from the trait-specific analyses. [b]Akaike Information Criterion (AIC) $= 2k - 2ln(L)$ where $2ln(L)$ is the logarithm of the maximum likelihood from the model and k is the number of model parameters in the model. [c]ΔAIC = AIC – AIC of the best model with the optimal α. The best model is red highlighted.

Supplementary Table 6: Inferring scaling factors in African ancestry cohort for LDAK-thin-α and GCTA-α models

Log-likelihood of African ancestry cohort (n=6179), LDAK-thin-α model

Traits	α= -1	α= -0.875	α= -0.75	α= -0.625	α= -0.50	α= -0.375	α= -0.25	α= -0.125	α= 0	α= 0.125
**BMI**	-2927.518	-2927.668	-2928.253	-2929.404	-2930.789	-2931.979	-2932.837	-2933.418	-2933.816	-2934.106
**Standing Height**	-2937.988	-2934.909	-2931.289	-2928.199	-2926.395	-2925.744	-2925.730	-2925.971	-2926.284	-2926.597
**Waist circumference**	-2939.807	-2939.515	-2939.597	-2940.323	-2941.455	-2942.544	-2943.381	-2943.977	-2944.402	-2944.721
**Hip circumference**	-2936.153	-2935.935	-2936.247	-2937.274	-2938.686	-2939.993	-2940.985	-2941.686	-2942.186	-2942.561
**Waist-hip ratio**	-2949.119	-2949.296	-2949.528	-2949.667	-2949.053	-2948.424	-2947.938	-2947.602	-2947.379	-2947.233
**Weight**	-2935.594	-2934.865	-2934.475	-2934.471	-2935.076	-2935.818	-2936.457	-2936.946	-2937.317	-2937.613
**Multivariate model[a]**	**-17626.179**	**-17622.2**	**-17619.4**	**-17619.3**	**-17621.5**	**-17624.5**	**-17627.3**	**-17629.6**	**-17631.4**	**-17632.8**
**AIC[b]**	**35264.358**	**35256.38**	**35250.78**	**35250.68**	**35254.91**	**35261**	**35266.66**	**35271.2**	**35274.77**	**35277.66**
**ΔAIC[c]**	**13.682**	**5.7**	**0.102**	**0**	**4.232**	**10.328**	**15.98**	**20.524**	**24.092**	**26.986**

Log-likelihood of African ancestry cohort (n=6179), GCTA-α model

Traits	α= -1	α= -0.875	α= -0.75	α= -0.625	α= -0.50	α= -0.375	α= -0.25	α= -0.125	α= 0	α= 0.125
**BMI**	-2927.832	-2928.195	-2929.163	-2930.665	-2932.198	-2933.397	-2934.226	-2934.791	-2935.189	-2935.490
**Standing Height**	-2937.849	-2934.538	-2930.892	-2928.063	-2926.565	-2926.078	-2926.105	-2926.334	-2926.617	-2926.899
**Waist circumference**	-2939.803	-2939.923	-2940.618	-2941.887	-2943.268	-2944.381	-2945.165	-2945.704	-2946.089	-2946.380
**Hip circumference**	-2937.302	-2937.341	-2938.024	-2939.333	-2940.798	-2942.017	-2942.904	-2943.531	-2943.989	-2944.343
**Waist-hip ratio**	-2949.289	-2949.363	-2949.555	-2949.763	-2949.447	-2949.134	-2948.896	-2948.734	-2948.629	-2948.563
**Weight**	-2936.051	-2935.517	-2935.394	-2935.875	-2936.695	-2937.496	-2938.133	-2938.616	-2938.991	-2939.297
**Multivariate model[a]**	**-17628.126**	**-17624.9**	**-17623.6**	**-17625.6**	**-17629**	**-17632.5**	**-17635.4**	**-17637.7**	**-17639.5**	**-17640.97**
**AIC[b]**	**35268.252**	**35261.75**	**35259.29**	**35263.17**	**35269.94**	**35277.01**	**35282.86**	**35287.42**	**35291.01**	**35293.94**
**ΔAIC[c]**	**8.96**	**2.462**	**0**	**3.88**	**10.65**	**17.714**	**23.566**	**28.128**	**31.716**	**34.652**

[a]Multivariate linear mixed model was used to get the log-likelihood of the scaling factor where residual and genetic correlations between traits were fixed as zero, i.e. the log-likelihood of this multivariate linear mixed model is the sum of log-likelihood values from the trait-specific analyses. [b]Akaike Information Criterion $(\text{AIC}) = 2k - 2ln(L)$ where $2\ln(L)$ is the logarithm of the maximum likelihood from the model and k is the number of model parameters in the model. [c]ΔAIC = AIC – AIC of the best model with the optimal α. The best model is red highlighted.

Supplementary Table 7: Inferring scaling factors in mixed ancestry cohort for LDAK-thin-α and GCTA-α models

Log-likelihood of Mixed ancestry cohort (n=11979), LDAK-thin-α model

Traits	α= -1	α= -0.875	α= -0.75	α= -0.625	α= -0.50	α= -0.375	α= -0.25	α= -0.125	α= 0	α= 0.125
**BMI**	-5588.259	-5585.895	-5588.577	-5594.217	-5599.984	-5604.549	-5607.815	-5610.084	-5611.664	-5612.583
**Standing Height**	-5596.967	-5580.956	-5569.985	-5565.634	-5565.784	-5567.876	-5570.469	-5573.009	-5575.333	-5577.420
**Waist circumference**	-5667.104	-5664.900	-5665.788	-5668.692	-5671.871	-5674.420	-5676.215	-5677.407	-5678.176	-5678.659
**Hip circumference**	-5660.905	-5659.907	-5661.853	-5665.352	-5668.692	-5671.190	-5672.886	-5674.006	-5674.749	-5675.251
**Waist-hip ratio**	-5686.903	-5684.125	-5682.170	-5681.575	-5681.901	-5682.573	-5683.274	-5683.889	-5684.399	-5684.811
**Weight**	-5641.025	-5636.521	-5636.154	-5638.865	-5642.432	-5645.546	-5647.911	-5649.638	-5650.909	-5651.872
**Multivariate model[a]**	**-33841.163**	**-33812.304**	**-33804.527**	**-33814.335**	**-33830.664**	**-33846.154**	**-33858.57**	**-33868.033**	**-33875.23**	**-33880.596**
**AIC[b]**	**67694.326**	**67636.608**	**67621.054**	**67640.67**	**67673.328**	**67704.308**	**67729.14**	**67748.066**	**67762.46**	**67773.192**
**ΔAIC[c]**	**73.272**	**15.554**	**0**	**19.616**	**52.274**	**83.254**	**108.086**	**127.012**	**141.406**	**152.138**

Log-likelihood of Mixed ancestry cohort (n= 11979), GCTA-α model

Traits	α= -1	α= -0.875	α= -0.75	α= -0.625	α= -0.50	α= -0.375	α= -0.25	α= -0.125	α= 0	α= 0.125
**BMI**	-5590.849	-5590.816	-5594.943	-5600.645	-5605.712	-5609.472	-5612.091	-5613.901	-5615.172	-5616.089
**Standing Height**	-5593.909	-5579.833	-5571.487	-5568.877	-5569.532	-5571.411	-5573.556	-5575.638	-5577.569	-5579.339
**Waist circumference**	-5667.672	-5667.488	-5669.858	-5673.188	-5676.094	-5678.174	-5679.547	-5680.424	-5680.975	-5681.317
**Hip circumference**	-5661.356	-5661.804	-5664.812	-5668.569	-5671.682	-5673.851	-5675.278	-5676.214	-5676.843	-5677.280
**Waist-hip ratio**	-5687.339	-5685.519	-5684.519	-5684.413	-5684.776	-5685.260	-5685.714	-5686.097	-5686.408	-5686.658
**Weight**	-5641.291	-5638.975	-5640.409	-5643.792	-5647.192	-5649.854	-5651.777	-5653.159	-5654.184	-5654.978
**Multivariate model[a]**	**-33842.416**	**-33824.435**	**-33826.028**	**-33839.484**	**-33854.988**	**-33868.022**	**-33877.963**	**-33885.433**	**-33891.151**	**-33895.661**
**AIC[b]**	**67696.832**	**67660.87**	**67664.056**	**67690.968**	**67721.976**	**67748.044**	**67767.926**	**67782.866**	**67794.302**	**67803.322**
**ΔAIC[c]**	**35.962**	**0**	**3.186**	**30.098**	**61.106**	**87.174**	**107.056**	**121.996**	**133.432**	**142.452**

[a]Multivariate linear mixed model was used to get the log-likelihood of the scaling factor where residual and genetic correlations between traits were fixed as zero, i.e. the log-likelihood of this multivariate linear mixed model is the sum of log-likelihood values from the trait-specific analyses. [b]Akaike Information Criterion $(\text{AIC}) = 2k - 2\ln(L)$ where $2ln(L)$ is the logarithm of the maximum likelihood from the model and k is the number of model parameters in the model. [c]ΔAIC = AIC – AIC of the best model with the optimal α. The best model is red highlighted.

Supplementary Table 8: Difference (AIC of LDAK-thin-α – AIC of GCTA-α) between two models

	Differences of Log-likelihood between of two model										
**Ancestry**	**α= -1**	**α= -0.875**	**α= -0.75**	**α= -0.625**	**α= -0.50**	**α= -0.375**	**α= -0.25**	**α= -0.125**	**α= 0**	**α= 0.125**	**Best α and model**
**White British**	-	-	-	150.942	173.182	192.154	206.024	215.024	220.104	222.256	**-0.25, GCTA-α**
**Other European**	-	-	-	109.138	111.692	115.714	118.9	120.646	121.098	120.58	**-0.125, GCTA-α**
**South Asian**	22.462	17.332	9.696	2.472	-2.782	-6.09	-8.024	-9.074	-9.654	-9.744	**-0.625, GCTA-α**
**African**	-7.622	-9.742	-9.216	-13.566	-14.638	-14.032	-13.464	-13.304	-13.422	-13.69	**-0.625, LDAK-thin-α**
**Mixed ancestry**	-2.506	-24.262	-43.002	-50.298	-48.648	-43.736	-38.786	-34.8	-31.842	-30.13	**-0.75, LDAK-thin-α**

Positive value (blue) indicates GCTA-$\boldsymbol{\alpha}$ is better over LDAK-thin-$\boldsymbol{\alpha}$ and negative value indicates LDAK-thin-$\boldsymbol{\alpha}$ is better.

Supplementary Table 9: Estimated heritability (h^2) and cross-ancestry genetic correlations (r_g) from 4 existing methods when simulation and estimation models agree (α= -0.5 for all the ancestry group).

		Bivariate simulation combining White British and African ancestry cohort			Bivariate simulation combining White British and South Asian ancestry cohort			Bivariate simulation combining South Asian and African ancestry cohort		
**True r_g**	**GRM**	**White British (h^2)**	**African (h^2)**	**Genetic correlation (r_g)**	**White British (h^2)**	**South Asian (h^2)**	**Genetic correlation (r_g)**	**South Asian (h^2)**	**African (h^2)**	**Genetic correlation (r_g)**
**r_g = 0**	GRM1	0.547±0.011	0.543±0.011	-0.001±0.014	0.535±0.012	0.512±0.012	0.012±0.017	0.531±0/011	0.527±0.011	-0.002±0.018
	GRM2	0.471±0.010	0.502±0.011	-0.004±0.028	0.512±0.011	0.492±0.012	-0.024±0.028	0.479±0.010	0.489±0.01	0.020±0.029
	GRM3	0.537±0.013	0.497±0.012	0.002±0.029	0.514±0.013	0.495±0.013	-0.040±0.031	0.515±0.012	0.484±0.012	0.021±0.038
	GRM4	0.494±0.011	0.482±0.011	-0.005±0.033	0.513±0.012	0.492±0.012	-0.004±0.045	0.491±0.011	0.477±0.012	0.020±0.041
**r_g = 0.25**	GRM1	0.531±0.010	0.540±0.010	0.152±0.013	0.528±0.012	0.485±0.011	0.171±0.019	0.531±0.010	0.528±0.011	0.162±0.015
	GRM2	0.471±0.010	0.502±0.011	0.250±0.027	0.491±0.011	0.489±0.011	0.235±0.025	0.480±0.010	0.506±0.012	0.251±0.026
	GRM3	0.529±0.013	0.496±0.012	0.246±0.049	0.513±0.013	0.482±0.012	0.239±0.049	0.527±0.012	0.487±0.012	0.252±0.060
	GRM4	0.492±0.011	0.482±0.011	0.273±0.047	0.493±0.012	0.480±0.012	0.265±0.038	0.495±0.011	0.484±0.011	0.255±0.039
**r_g = 0.50**	GRM1	0.525±0.009	0.539±0.010	0.251±0.011	0.529±0.011	0.502±0.011	0.299±0.011	0.518±0.010	0.535±0.010	0.284±0.013
	GRM2	0.469±0.010	0.515±0.011	0.454±0.023	0.504±0.011	0.485±0.011	0.431±0.026	0.475±0.010	0.515±0.011	0.470±0.025
	GRM3	0.527±0.013	0.512±0.013	0.464±0.051	0.525±0.013	0.484±0.013	0.456±0.052	0.494±0.013	0.489±0.012	0.472±0.036
	GRM4	0.486±0.011	0.488±0.011	0.498±0.036	0.502±0.011	0.482±0.012	0.477±0.048	0.478±0.011	0.488±0.011	0.511±0.046
**r_g = 0.75**	GRM1	0.519±0.008	0.533±0.008	0.325±0.010	0.524±0.010	0.508±0.009	0.419±0.010	0.530±0.010	0.538±0.009	0.371±0.010
	GRM2	0.473±0.009	0.518±0.010	0.661±0.019	0.511±0.011	0.496±0.011	0.670±0.019	0.481±0.010	0.517±0.011	0.661±0.020
	GRM3	0.511±0.013	0.511±0.012	0.712±0.051	0.526±0.014	0.492±0.012	0.726±0.042	0.516±0.013	0.496±0.013	0.728±0.045
	GRM4	0.486±0.011	0.483±0.011	0.764±0.049	0.507±0.012	0.489±0.011	0.746±0.055	0.485±0.012	0.482±0.012	0.765±0.049
**r_g = 1**	GRM1	0.535±0.007	0.529±0.006	0.375±0.010	0.524±0.008	0.503±0.007	0.441±0.008	0.511±0.007	0.527±0.008	0.428±0.011
	GRM2	0.492±0.010	0.518±0.010	0.851±0.021	0.511±0.011	0.494±0.010	0.891±0.015	0.477±0.009	0.517±0.010	0.810±0.013
	GRM3	0.530±0.012	0.512±0.014	0.990±0.065	0.512±0.013	0.481±0.012	0.980±0.049	0.497±0.013	0.512±0.013	0.968±0.070
	GRM4	0.503±0.011	0.508±0.010	0.978±0.049	0.498±0.012	0.479±0.012	1.020±0.052	0.488±0.011	0.481±0.011	0.979±0.042

In our simulations, we used three combinations for estimating cross-ancestry genetic correlation (White British vs. South Asian, White British vs. African and South Asian vs. African ancestry cohorts). In each ancestry group, we used 500,000 SNPs that were randomly selected from HapMap3 SNPs after QC. All the heritability and genetic correlation estimations were based on 500 replicates. To simulate phenotypes, we selected a random set of 1,000 SNPs as causal variants, which were presented for both ancestry groups. We used α = -0.5 when scaling the causal effects by ancestry-specific allele frequency in each ancestry group. Various values of genetic correlation were considered (0, 0.25, 0.50, 0.75 and 1.0). In the estimation, the four methods (GRM1 – 4) used α = -0.5 (standard scale factor in GRM estimation). GRM1 and 3 used all available SNPs from both ancestry groups (791581, 812332 and 777894, respectively for each combination) whereas GRM2 and 4 used only the set of SNPs common between two ancestry groups (208419, 187668 and 222106 for a, b, and c). When scaled with α = -0.5, GRM1 and 2 used allele frequency averaged between two ancestry groups whereas GRM3 and 4 used ancestry-specific allele frequency estimated from each ancestry group. Values highlighted in red indicate biased estimation at 5% level of significance.

Supplementary Table 10: Estimates of heritability (h^2) and cross-ancestry genetic correlations (r_g) from 4 existing methods when varying α values across ancestry groups.

		Bivariate simulation combining White British and African ancestry cohort			Bivariate simulation combining White British and South Asian ancestry cohort			Bivariate simulation combining South Asian and African ancestry cohort		
**True r_g**	**GRM**	**White British (h^2)**	**African (h^2)**	**Genetic correlation (r_g)**	**White British (h^2)**	**South Asian (h^2)**	**Genetic correlation (r_g)**	**South Asian (h^2)**	**African (h^2)**	**Genetic correlation (r_g)**
**r_g = 0**	GRM1	0.568±0.011	0.645±0.012	0.0002±0.013	0.559±0.013	0.479±0.011	0.026±0.017	0.510±0.011	0.615±0.011	0.0003±0.016
	GRM2	0.496±0.010	0.504±0.011	0.001±0.030	0.516±0.012	0.457±0.011	0.033±0.031	0.459±0.010	0.501±0.011	-0.013±0.028
	GRM3	0.552±0.012	0.671±0.011	0.004±0.022	0.538±0.013	0.459±0.012	0.035±0.039	0.487±0.012	0.636±0.012	-0.004±0.041
	GRM4	0.513±0.011	0.636±0.011	0.001±0.028	0.518±0.012	0.455±0.012	0.030±0.035	0.463±0.011	0.603±0.011	-0.002±0.053
**r_g = 0.25**	GRM1	0.584±0.012	0.647±0.011	0.133±0.012	0.569±0.012	0.482±0.011	0.164±0.016	0.505±0.011	0.617±0.011	0.200±0.025
	GRM2	0.512±0.010	0.503±0.010	0.228±0.024	0.533±0.012	0.456±0.010	0.241±0.027	0.465±0.010	0.507±0.012	0.229±0.025
	GRM3	0.567±0.013	0.670±0.011	0.185±0.028	0.551±0.013	0.466±0.012	0.248±0.048	0.512±0.012	0.627±0.013	0.206±0.026
	GRM4	0.526±0.011	0.631±0.010	0.210±0.030	0.529±0.012	0.459±0.010	0.251±0.036	0.484±0.011	0.600±0.012	0.215±0.031
**r_g = 0.50**	GRM1	0.574±0.011	0.621±0.011	0.193±0.011	0.551±0.011	0.487±0.010	0.285±0.012	0.507±0.010	0.607±0.011	0.239±0.013
	GRM2	0.507±0.011	0.516±0.011	0.364±0.024	0.534±0.011	0.463±0.011	0.449±0.034	0.446±0.009	0.510±0.011	0.389±0.041
	GRM3	0.565±0.013	0.657±0.011	0.345±0.033	0.541±0.013	0.459±0.013	0.464±0.051	0.498±0.012	0.633±0.011	0.378±0.039
	GRM4	0.513±0.012	0.628±0.010	0.374±0.040	0.529±0.012	0.454±0.012	0.491±0.051	0.465±0.010	0.601±0.011	0.401±0.029
**r_g = 0.75**	GRM1	0.556±0.009	0.581±0.010	0.271±0.009	0.546±0.010	0.487±0.009	0.381±0.009	0.496±0.009	0.595±0.010	0.357±0.010
	GRM2	0.508±0.010	0.535±0.010	0.551±0.019	0.521±0.011	0.463±0.011	0.641±0.022	0.442±0.009	0.512±0.010	0.611±0.019
	GRM3	0.547±0.012	0.687±0.011	0.543±0.031	0.544±0.013	0.453±0.013	0.731±0.056	0.468±0.012	0.633±0.011	0.622±0.036
	GRM4	0.513±0.011	0.656±0.010	0.582±0.036	0.512±0.012	0.454±0.013	0.751±0.039	0.446±0.011	0.605±0.011	0.684±0.042
**r_g = 1**	GRM1	0.554±0.008	0.585±0.008	0.325±0.010	0.538±0.008	0.486±0.007	0.436±0.010	0.506±0.009	0.585±0.009	0.480±0.012
	GRM2	0.523±0.010	0.513±0.010	0.635±0.017	0.536±0.010	0.465±0.010	0.796±0.019	0.465±0.009	0.508±0.010	0.709±0.017
	GRM3	0.567±0.013	0.656±0.011	0.701±0.034	0.540±0.013	0.451±0.013	0.961±0.067	0.497±0.012	0.630±0.011	0.740±0.048
	GRM4	0.531±0.011	0.627±0.011	0.781±0.055	0.521±0.012	0.447±0.012	1.003±0.054	0.461±0.011	0.604±0.011	0.801±0.037

In our simulation, we used three combinations for estimating cross-ancestry genetic correlation (White British vs. South Asian, White British vs. African and South Asian vs. African ancestry cohorts). In each ancestry group, we used 500,000 SNPs that were randomly selected from HapMap3 SNPs after QC. All the heritability and genetic correlation estimations were based on 500 replicates. To simulate phenotypes, we selected a random set of 1,000 SNPs as causal variants, which were presented for both ancestry groups. We used various α values that were specific to ancestries (α = -0.25, -0.625 and -0.75 for White British, South Asian and African ancestry cohorts, respectively) when scaling the causal effects by ancestry-specific allele frequency in each ancestry group. Various values of genetic correlation were considered (0, 0.25, 0.50, 0.75 and 1.0). In the estimation, we used existing methods (GRM1 – 4) that used the standard scale factor α = -0.5 in GRM estimation. GRM1 and 3 used all available SNPs from both ancestry groups (791581, 812332 and 777894, respectively for each combination) whereas GRM2 and 4 used only the set of SNPs common between two ancestry groups (208419, 187668 and 222106 for a, b, and c). When scaled with α = -0.5, GRM1 and 2 used allele frequency averaged between two ancestry groups whereas GRM3 and 4 used ancestry-specific allele frequency estimated from each ancestry group. Values highlighted in red indicate biased estimation at 5% level of significance.

Supplementary Table 11: **Estimated heritability (h^2) and cross-ancestry genetic correlations (r_g) from the proposed method when varying α values across ancestry groups.**

	Bivariate simulation combining White British and African ancestry cohort		
**True r_g**	**h^2 (British)**	**h^2 (African)**	**Estimated cross-ancestry r_g**
r_g = 0	0.499±0.011	0.497±0.008	0.005±0.029
r_g = 0.25	0.486±0.011	0.497±0.008	0.267±0.031
r_g = 0.50	0.494±0.011	0.495±0.009	0.489±0.034
r_g = 0.75	0.498±0.010	0.493±0.009	0.791±0.035
r_g = 1.0	0.501±0.011	0.508±0.008	1.061± 0.044
	**Bivariate simulation combining White British and South Asian ancestry cohort**		
**True r_g**	**h^2 (British)**	**h^2 (South Asian)**	**Estimated cross-ancestry r_g**
r_g = 0	0.501±0.012	0.486±0.013	0.017± 0.042
r_g = 0.25	0.498±0.011	0.485±0.013	0.261±0.030
r_g = 0.50	0.496±0.011	0.485±0.013	0.520± 0.044
r_g = 0.75	0.482±0.011	0.489±0.013	0.764±0.047
r_g = 1.0	0.489±0.012	0.511±0.012	1.050± 0.05
	**Bivariate simulation combining South Asian and African ancestry cohort**		
**True r_g**	**h^2 (South Asian)**	**h^2 (African)**	**Estimated cross-ancestry r_g**
r_g = 0	0.497±0.012	0.480±0.013	0.021± 0.039
r_g = 0.25	0.513±0.012	0.489±0.012	0.281± 0.040
r_g = 0.50	0.484±0.012	0.505±0.012	0.474± 0.048
r_g = 0.75	0.488±0.012	0.483±0.012	0.776±0.046
r_g = 1.0	0.505±0.012	0.487±0.013	0.982± 0.051

In our simulation, we used three combinations for estimating cross-ancestry genetic correlation (White British vs. South Asian, White British vs. African and South Asian vs. African ancestry cohorts). In each ancestry group, we used 500,000 SNPs that were randomly selected from HapMap3 SNPs after QC. To simulate phenotypes, we selected a random set of 1,000 SNPs as causal variants, which were presented for both ancestry groups. We used various α values that were specific to ancestries (α = -0.25, -0.625 and -0.75 for White British, South Asian and African ancestry cohorts, respectively) when scaling the causal effects by ancestry-specific allele frequency in each ancestry group. Various values of genetic correlation were considered (0, 0.25, 0.50, 0.75 and 1.0). In the estimation, we applied the proposed method that used ancestry-specific α value and ancestry-specific allele frequency in GRM estimation. GRM was estimated based common SNP between population (208419, 187668 and 222106, respectively for each combination) and was implemented MTG2-*software* [1].

Supplementary Table 12: **Estimates of heritability (h^2) and cross-ancestry genetic correlations (r_g) from simulated data using estimated α and proposed approach of GRM across ancestry groups (true h^2 = 0.5 and r_g = 1)**

**Bivariate simulation combining White British and African ancestry cohort**			
**Number of causal SNP**	**Estimated h_1^2 (British) (True h_1^2= 0.5)**	**Estimated h_2^2 (African) (True h_2^2= 0.5)**	**Estimated cross-ancestry r_g (True r_g = 1.0)**
100	0.487±0.012	0.509±0.011	1.031±0.050
1000	0.501±0.011	0.508±0.008	1.061± 0.044
10000	0.489±0.012	0.505±0.009	1.021±0.041
100000	0.489±0.011	0.497±0.010	0.981±0.037
**Bivariate simulation combining White British and South Asian ancestry cohort**			
**Number of causal SNP**	**Estimated h_1^2 (British) (True h_1^2= 0.5)**	**Estimated h_2^2 (South Asian) (True h_2^2= 0.5)**	**Estimated cross-ancestry r_g (True r_g = 1.0)**
100	0.505±0.012	0.474±0.013	0.978±0.061
1000	0.489±0.012	0.511±0.012	1.050± 0.05
10000	0.499±0.011	0.503±0.012	1.023±0.042
100000	0.488±0.011	0.495±0.011	1.012±0.039
**Bivariate simulation combining South Asian and African ancestry cohort**			
**Number of causal SNP**	**Estimated h_1^2 (South Asian) (True h_1^2= 0.5)**	**Estimated h_2^2 (African) (True h_2^2= 0.5)**	**Estimated cross-ancestry r_g (True r_g = 1.0)**
100	0.487±0.011	0.488±0.010	0.984±0.058
1000	0.505±0.012	0.487±0.013	0.982± 0.051
10000	0.498±0.011	0.497±0.012	1.022±0.040
100000	0.485±0.012	0.501±0.011	0.991±0.038

Simulation was based on 100, 1000, 10000 and 100000 random common SNPs as causal and following estimated scaling factor (α) across ancestries (-0.25 for White British, -0.625 for South Asian and -0.75 for African ancestry cohort). All the heritability and genetic correlation estimations were based on 500 replicates. For simulation true heritability was 0.50 for both ancestry groups in each combined population. GRM was estimated based on our proposed approach and was implemented MTG2[1].

Supplementary Table 13: Percentage of the causal SNPs that are common between the two ancestries in the simulation when applying MAF QC differently.

**Pairs of ancestries**	**Percentage of causal SNPs common between two ancestries**	
	**MAF <0.01 (QC) applied for each ancestry**	**MAF <0.05 (QC) applied for each ancestry**
White British vs Other European	96.52	89.95
White British vs Asian	95.07	81.23
Other European vs Asian	94.44	81.28
Asian vs African	91.78	72.04
White British vs African	90.48	69.64
Other European vs African	89.80	69.65

The simulation was based on the real genotypes, using ancestry-specific alphas (estimated from the real data as shown in Figure 1). The sample size used in this simulation was 5,000 for each ancestry (total 10,000). After QC including MAF < 0.001, the number of SNPs was ~200,000 across ancestry pairs, among which 10,000 SNPs were selected as causal. For the genotypic data, QC of MAF < 0.01 or MAF < 0.05 was applied for each ancestry to check what % of the causal SNPs are common between the two ancestries of each pair.

Supplementary Table 14: Comparing heritability and cross ancestry genetic correlation Popcorn, XPASS and Proposed method using estimated scale factor

**True values**		**Model using estimated scaling factor (α = -0.25, -0.625 and -0.75 respectively, for White British, Asian and African)**								
	**Combinations of ethnicities**	**Popcorn**			**XPASS**			**Proposed method**		
		**Estimated h^2_{eth1}**	**Estimated h^2_{eth2}**	**Estimated r_g**	**Estimated h^2_{eth1}**	**Estimated h^2_{eth2}**	**Estimated r_g**	**Estimated h^2_{eth1}**	**Estimated h^2_{eth2}**	**Estimated r_g**
h^2_{eth1}= 0.5 h^2_{eth2}= 0.5 r_g = 0.5	White British and African	0.46±0.016	0.13±0.019	0.36±0.051	0.53±0.011	0.65±0.069	0.55±0.028	0.497±0.008	0.508±0.008	0.505±0.018
	White British and Asian	0.47±0.016	0.37±0.022	0.41±0.044	0.55±0.010	0.46±0.023	0.54±0.017	0.496±0.008	0.501±0.009	0.510±0.013
	Asian and African	0.41±0.014	0.31±0.025	0.40±0.049	0.50±0.043	0.53±0.067	0.58±0.034	0.495±0.008	0.494±0.009	0.514±0.019

Simulation was based on 1,000 random common SNPs as causal and 10,000 individuals (5000 from each ancestry). The reported values are the average of the estimated heritability and cross-ancestry genetic correlation based on 50 replications. h^2_{eth1} and h^2_{eth1} indicates the estimated heritability of first and second ancestries. The true heritability was simulated as 0.5 for both ancestry groups and the true genetic correlation was simulated as 0.50 for each pair of ancestries. Red indicates biased estimations.

Supplementary Table 15: Comparison of the computing time and capacity among Popcorn, XPASS and our proposed method

Conditions	Popcorn	XPASS	Proposed method
(1000+1000) individual and ~200k common SNP	For score: 1 min 46 sec<br>For GWAS: 30sec<br>For estimation: 39 sec<br><br>RAM: 0.34GB	For GWAS: 30 sec<br>For estimation: 4 min 19 sec<br><br>RAM: 16GB	For GRM: 1 min 37 sec<br>For GREML: 15 sec<br><br>RAM: 0.93GB
(5000+5000) individual and ~200k common SNP	For score: 4min 31 sec<br>For GWAS: 2 min<br>For estimation: 42 sec<br><br>RAM: 0.36 GB	For GWAS: 2 min<br>For estimation: 26 min<br><br>RAM: 54GB	For GRM: 17 min 59 sec<br>For GREML: 4 min 21 sec<br><br>RAM: 22GB
(10000+10000) individual and ~200k common SNP	For score: 7 min 39 sec<br>For GWAS: 2 min 12 sec<br>For estimation: 45 sec<br><br>RAM: 0.37GB	For GWAS: 2 min 12 sec<br>For estimation: 1 hr 22 min<br><br>RAM: 76GB	For GRM: 58 min 05 sec<br>For GREML: 27 min 9 sec<br><br>RAM: 52GB

Using a single CPU (2.0 GHz).
Proposed method and XPASS can use a parallel computing and their computational efficiency can be further increased. Parallel computing is not available for Popcorn.

Supplementary Table 16: Computational efficiency of the proposed method with parallel computing

Conditions	Without -thread	-thread 10	-thread 20	-thread 50
	**For GRM**			
(10000+10000) individual and ~200k common SNP	Reading plink file:3 min 5sec<br>Estimate GRM:38 min 29 sec<br>Record GRM: 16 min 41	Reading plink file: 3 min 3 sec<br>Estimate GRM:3 min 46 sec<br>Record GRM:16 min 4 sec	Reading plink file:3 min 1 sec<br>Estimate GRM: 2 min 3 sec<br>Record GRM: 15 min 58 sec	Reading plink file: 3 min<br>Estimate GRM: 1 min 24 sec<br>Record GRM: 15 min 55 sec
	**For GREML**			
	Reading file: 10 min 3 sec<br>For estimation: 17 min 7 sec	Reading file: 10 min 1 sec<br>For estimation: 3 min 15 sec	Reading file: 10 min 0 sec<br>For estimation: 2 min 16	Reading file: 10 min 3 sec<br>For estimation: 1 min 36 sec

For estimation of GRM and estimation of genetic parameters by GREML, the computational efficiency increases with parallel computing. RAM is not changed with parallel computing, therefore not shown here (see Supplementary Table 15). Note that reading and recording are not computationally paralleled in the current version.

Supplementary Table 17: Estimated cross-ancestry genetic correlations (SE) for BMI

	White British (n=29,628)	Other European (n=25,909)	South Asian (n=5,719)	African (n=5,872)	Mixed ancestry (n=11,267)
**White British**		**1.081 (0.043)**<br>**P= 5.96e-02**<br>h_c^2 (WB)= 0.222 (0.012)<br>h_c^2 (OE)= 0.216 (0.013)	**0.869 (0.111)**<br>**P= 2.37e-01**<br>h_c^2 (WB)=0.195 (0.012)<br>h_c^2 (SAS)= 0.296 (0.055)	**0.672 (0.131)**<br>**P= 1.22e-02**<br>h_c^2 (WB)= 0.177 (0.011)<br>h_c^2 (AFR)= 0.245 (0.051)	**0.884 (0.082)**<br>**P= 1.57e-01**<br>h_c^2 (WB)= 0.160 (0.011)<br>h_c^2 (MA)= 0.272 (0.029)
**Other European**			**0.909 (0.112)**<br>**P= 4.16e-01**<br>h_c^2 (OE)= 0.199 (0.013)<br>h_c^2 (SAS)= 0.312 (0.056)	**0.549 (0.134)**<br>**P= 7.63e-04**<br>h_c^2 (OE)=0.178 (0.012)<br>h_c^2 (AFR)= 0.239 (0.051)	**0.913 (0.085)**<br>**P= 3.06e-01**<br>h_c^2 (OE)=0.158 (0.012)<br>h_c^2 (MA)=0.282 (0.029)
**South Asian**				**1.015 (0.260)**<br>**P= 9.53-01**<br>h_c^2 (SAS)=0.238 (0.051)<br>h_c^2 (AFR)= 0.276 (0.060)	**Cohort3 is the subset of cohort 6**
**African**					**0.699 (0.194)**<br>**P= 1.21e-01**<br>h_c^2 (AFR)= 0.187 (0.048)<br>h_c^2 (MA)= 0.203 (0.026)
**Mixed ancestry**					

P is the *p*-value based on Wald's test statistics for the null hypothesis of the estimated cross ancestry genetic correlation r_g=1 (i.e., a two-sided test). h_c^2 is the estimated SNP-based heritability from the bivariate GREML, using SNPs common between two ancestries. WB, OE, SAS, AFR and MA indicates White British, Other European, South Asian, African, and Mixed ancestry cohorts.

Supplementary Table 18: Estimated cross-ancestry genetic correlations (SE) for standing height

	White British (n=29,663)	Other European (n=25,940)	South Asian (n=5,725)	African (n=5,880)	Mixed ancestry (n=11,280)
**White British**		**1.010 (0.018)**<br>**P= 5.78e-01**<br>h_c^2 (WB)= 0.499 (0.011)<br>h_c^2 (OE)= 0.472 (0.012)	**0.904 (0.063)**<br>**P= 1.27e-01**<br>h_c^2 (WB)=0.473 (0.012)<br>h_c^2 (SAS)=0.458 (0.052)	**0.876 (0.118)**<br>**P= 2.93e-01**<br>h_c^2 (WB)= 0.446 (0.012)<br>h_c^2 (AFR)= 0.216 (0.047)	**1.006 (0.056)**<br>**P= 9.14e-01**<br>h_c^2 (WB)= 0.392 (0.011)<br>h_c^2 (MA)= 0.318 (0.028)
**Other European**			**0.847 (0.062)**<br>**P= 1.35e-02**<br>h_c^2 (OE)= 0.449 (0.0130)<br>h_c^2 (SAS)= 0.483 (0.054)	**0.877 (0.118)**<br>**P= 2.97e-01**<br>h_c^2 (OE)=0.426 (0.013)<br>h_c^2 (AFR)= 0.224 (0.047)	**0.979 (0.057)**<br>**P= 7.12e-01**<br>h_c^2 (OE)=0.365 (0.012)<br>h_c^2 (MA)= 0.334 (0.029)
**South Asian**				**0.356 (0.169)**<br>**P= 1.38e-04**<br>h_c^2 (SAS)=0.452 (0.053)<br>h_c^2 (AFR)= 0.219 (0.049)	**Cohort3 is the subset of cohort 6**
**African**					**0.512 (0.158)**<br>**P= 2.01e-03**<br>h_c^2 (AFR)=0.191 (0.046)<br>h_c^2 (MA)=0.273 (0.027)
**Mixed ancestry**					

P is the *p*-value based on Wald's test statistics for the null hypothesis of the estimated cross ancestry genetic correlation r_g=1 (i.e., a two-sided test). h_c^2 is the estimated SNP-based heritability from the bivariate GREML, using SNPs common between two ancestries. WB, OE, SAS, AFR and MA indicates White British, Other European, South Asian, African, and Mixed ancestry cohorts.

Supplementary Table 19: Estimated cross-ancestry genetic correlations (SE) for waist circumference

	White British (n=29,666)	Other European (n=25,946)	South Asian (n=5,807)	African (n=5,893)	Mixed ancestry (n=11,375)
**White British**		**1.056 (0.052)**<br>**P= 2.81e-01**<br>h_c^2 (WB)= 0.195 (0.0116)<br>h_c^2 (OE)= 0.178 (0.0126)	**0.908 (0.145)**<br>**P= 5.25e-01**<br>h_c^2 (WB)= 0.169 (0.0113)<br>h_c^2 (SAS)= 0.222 (0.0554)	**0.627 (0.144)**<br>**P= 9.58e-03**<br>h_c^2 (WB)= 0.149 (0.011)<br>h_c^2 (AFR)= 0.224 (0.050)	**0.936 (0.107)**<br>**P= 5.49e-01**<br>h_c^2 (WB)=0.135 (0.010)<br>h_c^2 (MA)= 0.201 (0.029)
**Other European**			**1.068 (0.161)**<br>**P=6.73e-01**<br>h_c^2 (OE)=0.159 (0.0124)<br>h_c^2 (SAS)=0.226 (0.0553)	**0.507 (0.148)**<br>**P= 8.65-04**<br>h_c^2 (OE)=0.145 (0.012)<br>h_c^2 (AFR)=0.224 (0.051)	**1.036 (0.117)**<br>**P= 7.57e-01**<br>h_c^2 (OE)= 0.124 (0.011)<br>h_c^2 (MA)= 0.207 (0.029)
**South Asian**				**1.299 (0.361)**<br>**P= 4.07e-01**<br>h_c^2 (SAS)= 0.158 (0.050)<br>h_c^2 (AFR)= 0.213 (0.051)	**Cohort3 is the subset of cohort 6**
**African**					**0.625 (0.256)**<br>**P= 1.42e-01**<br>h_c^2 (AFR)=0.174 (0.047)<br>h_c^2 (MA)= 0.136 (0.025)
**Mixed ancestry**					

P is the *p*-value based on Wald's test statistics for the null hypothesis of the estimated cross ancestry genetic correlation r_g=1 (i.e., a two-sided test). h_c^2 is the estimated SNP-based heritability from the bivariate GREML, using SNPs common between two ancestries. WB, OE, SAS, AFR and MA indicates White British, Other European, South Asian, African, and Mixed ancestry cohorts.

Supplementary Table 20: Estimated cross-ancestry genetic correlations (SE) for hip circumference

	White British (n=29,677)	Other European (n=25,946)	South Asian (n=5,807)	African (n=5,892)	Mixed ancestry (n=11,373)
**White British**		**1.076 (0.046)**<br>**P= 9.84e-02**<br>h_c^2 (WB)=0.214 (0.012)<br>h_c^2 (OE)= 0.201 (0.013)	**1.099 (0.187)**<br>**P= 5.96e-01**<br>h_c^2 (WB)=0.187 (0.011)<br>h_c^2 (SAS)=0.184 (0.055)	**0.778 (0.141)**<br>**P= 1.15e-01**<br>h_c^2 (WB)= 0.169 (0.011)<br>h_c^2 (AFR)= 0.230 (0.045)	**0.992 (0.097)**<br>**P= 9.35e-01**<br>h_c^2 (WB)=0.156 (0.011)<br>h_c^2 (MA)= 0.222 (0.029)
**Other European**			**1.027 (0.168)**<br>**P= 8.72e-01**<br>h_c^2 (OE)=0.182 (0.013)<br>h_c^2 (SAS)= 0.204 (0.056)	**0.572 (0.143)**<br>**P= 2.76e-03**<br>h_c^2 (OE)=0.166 (0.012)<br>h_c^2 (AFR)= 0.222 (0.049)	**0.956 (0.106)**<br>**P= 6.79e-01**<br>h_c^2 (OE)=0.143 (0.012)<br>h_c^2 (MA)= 0.213 (0.029)
**South Asian**				**1.391 (0.396)**<br>**P= 3.23e-01**<br>h_c^2 (SAS)=0.140 (0.051)<br>h_c^2 (AFR)= 0.217 (0.050)	**Cohort3 is the subset of cohort 6**
**African**					**0.721 (0.221)**<br>**P= 2.06e-01**<br>h_c^2 (AFR)=0.178 (0.047)<br>h_c^2 (MA)= 0.159 (0.026)
**Mixed ancestry**					

P is the *p*-value based on Wald's test statistics for the null hypothesis of the estimated cross ancestry genetic correlation r_g=1 (i.e., a two-sided test). h_c^2 is the estimated SNP-based heritability from the bivariate GREML, using SNPs common between two ancestries. WB, OE, SAS, AFR and MA indicates White British, Other European, South Asian, African, and Mixed ancestry cohorts.

Supplementary Table 21: Estimated cross-ancestry genetic correlations (SE) for waist-hip ratio

	White British (n=29,664)	Other European (n=25,941)	South Asian (n=5,807)	African (n=5,095)	Mixed ancestry (n=11,373)
**White British**		**1.049 (0.069)**<br>**P= 4.77e-01**<br>h_c^2 (WB)= 0.152 (0.011)<br>h_c^2 (OE)= 0.139 (0.012)	**0.765 (0.179)**<br>**P= 1.89e-01**<br>h_c^2 (WB)=0.135 (0.011)<br>h_c^2 (SAS)= 0.163 (0.057)	**NA**<br>h_c^2 (WB)= 0.118 (0.010)<br>h_c^2 (AFR)= -0.001 (0.024)	**0.977 (0.179)**<br>**P= 8.97e-01**<br>h_c^2 (WB)= 0.099 (0.010)<br>h_c^2 (MA)= 0.111 (0.028)
**Other European**			**0.921 (0.206)**<br>**P= 7.01e-01**<br>h_c^2 (OE)=0.127 (0.012)<br>h_c^2 (SAS)= 0.164 (0.057)	**NA**<br>h_c^2 (OE)=0.117 (0.011)<br>h_c^2 (AFR)= -0.017 (0.004)	**0.991 (0.189)**<br>**P= 9.60e-01**<br>h_c^2 (OE)=0.095 (0.011)<br>h_c^2 (MA)= 0.112 (0.028)
**South Asian**				**NA**<br>h_c^2 (SAS)=0.102 (0.050)<br>h_c^2 (AFR)= -0.007 (0.011)	**Cohort3 is the subset of cohort 6**
**African**					**NA**<br>h_c^2 (AFR)=-0.007 (0.015)<br>h_c^2 (MA)= 0.059 (0.023)
**Mixed ancestry**					

P is the *p*-value based on Wald's test statistics for the null hypothesis of the estimated cross ancestry genetic correlation r_g=1 (i.e., a two-sided test). h_c^2 is the estimated SNP-based heritability from the bivariate GREML, using SNPs common between two ancestries. WB, OE, SAS, AFR and MA indicates White British, Other European, South Asian, African, and Mixed ancestry cohorts. Some of the cross-ancestry genetic correlation was estimated as NA in the pairs involving African and this is because of no significant estimation of heritability in African ancestry cohorts.

Supplementary Table 22: Estimated cross-ancestry genetic correlations (SE) for weight

	White British (n=29,632)	Other European (n=25,919)	South Asian (n=5,802)	African (n=5,884)	Mixed ancestry (n=11,360)
**White British**		**1.062 (0.039)**<br>**P= 1.11e-01**<br>h_c^2 (WB)=0.2608 (0.0118)<br>h_c^2 (OE)=0.2351 (0.0129)	**0.891 (0.099)**<br>**P= 2.71e-01**<br>h_c^2 (WB)=0.2339 (0.0117)<br>h_c^2 (SAS)= 0.3235 (0.0549)	**0.832 (0.137)**<br>**P= 2.20e-01**<br>h_c^2 (WB)= 0.215 (0.011)<br>h_c^2 (AFR)= 0.223 (0.049)	**0.950 (0.077)**<br>**P= 5.16e-01**<br>h_c^2 (WB)=0.1926 (0.0108)<br>h_c^2 (MA)=0.2728 (0.0292)
**Other European**			**0.956 (0.101)**<br>**P= 6.63e-01**<br>h_c^2 (OE)=0.2171 (0.0128)<br>h_c^2 (SAS)=0.3486 (0.0549)	**0.624 (0.139)**<br>**P= 6.83e-03**<br>h_c^2 (OE)=0.197 (0.012)<br>h_c^2 (AFR)= 0.216 (0.049)	**0.957 (0.083)**<br>**P= 6.04e-01**<br>h_c^2 (OE)=0.1734 (0.0117)<br>h_c^2 (MA)=0.2765 (0.0294)
**South Asian**				**1.054 (0.254)**<br>**P= 8.31e-01**<br>h_c^2 (SAS)=0.259 (0.051)<br>h_c^2 (AFR)=0.223 (0.051)	**Cohort3 is the subset of cohort 6**
**African**					**0.670 (0.194)**<br>**P= 8.88e-02**<br>h_c^2 (AFR)=0.182 (0.047)<br>h_c^2 (MA)=0.204 (0.026)
**Mixed ancestry**					

P is the *p*-value based on Wald's test statistics for the null hypothesis of the estimated cross ancestry genetic correlation r_g=1 (i.e., a two-sided test). h_c^2 is the estimated SNP-based heritability from the bivariate GREML, using SNPs common between two ancestries. WB, OE, SAS, AFR and MA indicates White British, Other European, South Asian, African, and Mixed ancestry cohorts.

Supplementary Table 23: Determining trait specific scale factor for White British

**Log-likelihood**													
**Traits**	**-1**	**-0.875**	**-0.75**	**α= -0.625**	**α= -0.50**	**α= -0.375**	**α= -0.25**	**α= -0.125**	**α= 0**	**α= 0.125**	**α= 0.25**	**α= 0.375**	**α= 0.5**
**PR**	-13799.8	-13786.35	-13775.38	-13768.32	-13764.48	-13762.67	-13762.01	**-13761.96**	-13762.25	-13762.69	-13763.21	-13763.76	-13764.3
**BMR**	-14306.4	-14249.62	-14207.57	-14182.13	-14169.27	-14164.14	**-14163.26**	-14164.62	-14167.12	-14170.19	**-14173.51**	-14176.92	-14180.3
**WBFFM**	-14286.3	-14224.01	-14178.08	-14150.29	-14136.18	-14130.47	**-14129.4**	-14130.78	-14133.44	-14136.74	-14140.34	-14144.05	-14147.76
**BFP**	-14392.2	-14362.64	-14339.62	-14324.94	-14316.82	-14312.84	**-14311.24**	**-14310.97**	-14311.47	-14312.41	-14313.61	-14314.94	-14316.35
**EDU**	-14718.8	-14701.62	-14689.29	-14682.31	-14679.11	-14678.04	**-14678.04**	-14678.55	-14679.29	-14680.13	-14680.99	-14681.85	-14682.69
**Delta AIC**													
**Traits**	**-1**	**-0.875**	**-0.75**	**α= -0.625**	**α= -0.50**	**α= -0.375**	**α= -0.25**	**α= -0.125**	**α= 0**	**α= 0.125**	**α= 0.25**	**α= 0.375**	**α= 0.5**
**PR**	75.732	48.777	26.828	12.711	5.031	1.415	0.089	**0**	0.564	1.456	2.497	3.584	4.6614
**BMR**	286.285	172.704	88.612	37.726	12.004	1.746	**0**	2.717	7.722	13.859	20.503	27.305	34.068
**WBFFM**	313.897	189.212	97.361	41.784	13.554	2.134	**0**	2.768	8.081	14.681	21.882	29.299	36.711
**BFP**	162.477	103.337	57.286	27.926	11.697	3.741	0.533	**0**	0.995	2.874	5.2638	7.936	10.751
**EDU**	81.518	47.161	22.505	8.553	2.155	0.007	**0**	1.017	2.501	4.179	5.9132	7.634	9.306

PR= Pulse rate, BMR= Basal metabolic rate, WBFFM= Whole Body fat free mass, BFP= Body fat percentage, EDU= Education. Akaike Information Criterion $(\text{AIC}) = 2k - 2ln(L)$ where $\ln(L)$ is the logarithm of the maximum likelihood from the model and k is the number of model parameters in the model. ΔAIC = AIC – AIC of the best model with the optimal α. The best model is highlighted.

Supplementary Table 24: Determining trait specific scale factor for the Other Europeans

**Log-likelihood**													
**Traits**	**-1**	**-0.875**	**-0.75**	***α*= -0.625**	***α*= -0.50**	***α*= -0.375**	***α*= -0.25**	***α*= -0.125**	***α*= 0**	***α*= 0.125**	***α*= 0.25**	***α*= 0.375**	***α*= 0.5**
**PR**	-12092.82	-12083.76	-12076.32	-12071.44	-12068.62	-12067.07	-12066.24	-12065.81	-12065.6	**-12065.53**	-12065.55	-12065.62	-12065.74
**BMR**	-12542.48	-12500.99	-12468.03	-12446.66	-12434.73	-12428.91	-12426.68	**-12426.5**	-12427.5	-12429.17	-12431.23	-12433.5	-12435.86
**WBFFM**	-12514.78	-12468.83	-12433.03	-12410.3	-12397.97	-12392.27	**-12390.4**	-12390.67	-12392.13	-12394.28	-12396.8	-12399.52	-12402.31
**BFP**	-12582.75	-12559.5	-12540.82	-12528.61	-12521.65	-12518.08	-12516.52	**-12516.12**	-12516.39	-12517.07	-12517.99	-12519.05	-12520.2
**EDU**	-12813.73	-12802.24	-12792.89	-12786.71	-12783.14	-12781.22	-12780.25	-12779.81	**-12779.65**	-12779.67	-12779.79	-12779.97	-12780.19
**Delta AIC**													
**Traits**	**-1**	**-0.875**	**-0.75**	***α*= -0.625**	***α*= -0.50**	***α*= -0.375**	***α*= -0.25**	***α*= -0.125**	***α*= 0**	***α*= 0.125**	***α*= 0.25**	***α*= 0.375**	***α*= 0.5**
**PR**	54.581	36.448	21.573	11.814	6.166	3.072	1.416	0.554	0.142	**0**	0.029	0.177	0.408
**BMR**	231.951	148.962	83.054	40.318	16.451	4.816	0.358	**0**	1.991	5.336	9.453	13.987	18.719
**WBFFM**	248.763	156.861	85.267	39.793	15.138	3.747	**0**	0.532	3.467	7.758	12.799	18.229	23.824
**BFP**	133.263	86.760	49.404	24.979	11.060	3.929	0.805	**0**	0.552	1.91	3.750	5.877	8.169
**EDU**	68.159	45.181	26.464	14.117	6.971	3.137	1.194	0.303	**0**	0.034	0.270	0.632	1.075

PR= Pulse rate, BMR= Basal metabolic rate, WBFFM= Whole Body fat free mass, BFP= Body fat percentage, EDU= Education. Akaike Information Criterion $(\text{AIC}) = 2k - 2ln(L)$ where $\ln(L)$ is the logarithm of the maximum likelihood from the model and k is the number of model parameters in the model. ΔAIC = AIC – AIC of the best model with the optimal α. The best model is highlighted.

Supplementary Table 25: Determining trait specific scale factor for South Asians

Log-likelihood													
**Traits**	**-1**	**-0.875**	**-0.75**	***α*= -0.625**	***α*= -0.50**	***α*= -0.375**	***α*= -0.25**	***α*= -0.125**	***α*= 0**	***α*= 0.125**	***α*= 0.25**	***α*= 0.375**	***α*= 0.5**
**PR**	-2829.631	-2828.456	-2827.276	-2826.38	-2825.819	-2825.499	-2825.33	-2825.246	-2825.211	**-2825.203**	-2825.209	-2825.224	-2825.242
**BMR**	-2853.446	-2850.913	-2849.295	**-2848.67**	-2848.683	-2848.964	-2849.316	-2849.66	-2849.976	-2850.263	-2850.522	-2850.759	-2850.975
**WBFFM**	-2853.486	-2850.594	-2848.569	-2847.58	**-2847.298**	-2847.371	-2847.576	-2847.817	-2848.06	-2848.294	-2848.516	-2848.725	-2848.921
**BFP**	-2861.989	-2860.741	-2860.053	**-2859.94**	-2860.182	-2860.567	-2860.979	-2861.374	-2861.734	-2862.059	-2862.35	-2862.612	-2862.847
**EDU**	-2915.38	-2913.514	-2912.449	**-2912.14**	-2912.301	-2912.693	-2913.17	-2913.665	-2914.148	-2914.605	-2915.031	-2915.423	-2915.78
**Delta AIC**													
**Traits**	**-1**	**-0.875**	**-0.75**	***α*= -0.625**	***α*= -0.50**	***α*= -0.375**	***α*= -0.25**	***α*= -0.125**	***α*= 0**	***α*= 0.125**	***α*= 0.25**	***α*= 0.375**	***α*= 0.5**
**PR**	8.856	6.506	4.147	2.360	1.233	0.593	0.254	0.087	0.017	**0**	0.013	0.042	0.079
**BMR**	9.548	4.483	1.247	**0**	0.023	0.585	1.288	1.977	2.609	3.182	3.701	4.174	4.606
**WBFFM**	12.376	6.591	2.540	0.555	**0**	0.146	0.555	1.037	1.523	1.991	2.435	2.853	3.245
**BFP**	4.096	1.598	0.224	**0**	0.482	1.250	2.076	2.864	3.585	4.235	4.817	5.340	5.812
**EDU**	6.484	2.754	0.623	**0**	0.328	1.111	2.065	3.054	4.020	4.935	5.787	6.571	7.285

PR= Pulse rate, BMR= Basal metabolic rate, WBFFM= Whole Body fat free mass, BFP= Body fat percentage, EDU= Education. Akaike Information Criterion $(\text{AIC}) = 2k - 2ln(L)$ where $\ln(L)$ is the logarithm of the maximum likelihood from the model and k is the number of model parameters in the model. ΔAIC = AIC – AIC of the best model with the optimal α. The best model is highlighted.

Supplementary Table 26: Determining trait specific scale factor for Africans

Log-likelihood													
**Traits**	**-1**	**-0.875**	**-0.75**	**α= -0.625**	**α= -0.50**	**α= -0.375**	**α= -0.25**	**α= -0.125**	**α= 0**	**α= 0.125**	**α= 0.25**	**α= 0.375**	**α= 0.5**
**PR**	-2900.899	-2900.424	-2899.894	-2899.46	-2899.203	**-2899.062**	-2899.066	-2899.085	-2899.129	-2899.186	-2899.249	-2899.313	-2899.376
**BMR**	-2883.581	-2881.622	-2879.62	-2878.241	-2877.661	**-2877.585**	-2877.711	-2877.896	-2878.092	-2878.286	-2878.475	-2878.661	-2878.841
**WBFFM**	-2883.513	-2881.409	-2879.029	-2877.097	-2875.952	-2875.406	-2875.175	-2875.093	**-2875.085**	-2875.123	-2875.19	-2875.278	-2875.38
**BFP**	**-2873.264**	-2873.593	-2874.651	-2876.324	-2878.025	-2879.345	-2880.254	-2880.868	-2881.298	-2881.615	-2881.861	-2882.061	-2882.227
**EDU**	-2963.444	**-2962.38**	-2962.797	-2964.559	-2966.684	-2968.453	-2969.728	-2970.614	-2971.238	-2971.69	-2972.026	-2972.284	-2972.485
**Delta AIC**													
**Traits**	**-1**	**-0.875**	**-0.75**	**α= -0.625**	**α= -0.50**	**α= -0.375**	**α= -0.25**	**α= -0.125**	**α= 0**	**α= 0.125**	**α= 0.25**	**α= 0.375**	**α= 0.5**
**PR**	3.675	2.724	1.664	0.797	0.283	**0**	0.008	0.047	0.134	0.248	0.374	0.502	0.628
**BMR**	11.991	8.075	4.071	1.312	0.153	**0**	0.253	0.623	1.014	1.401	1.781	2.152	2.512
**WBFFM**	16.856	12.647	7.887	4.022	1.734	0.642	0.18	0.015	**0**	0.075	0.209	0.385	0.589
**BFP**	**0**	0.658	2.773	6.12	9.521	12.161	13.979	15.208	16.068	16.702	17.194	17.594	17.927
**EDU**	2.128	**0**	0.834	4.358	8.609	12.147	14.696	16.468	17.717	18.619	19.293	19.808	20.211

PR= Pulse rate, BMR= Basal metabolic rate, WBFFM= Whole Body fat free mass, BFP= Body fat percentage, EDU= Education. Akaike Information Criterion $(\text{AIC}) = 2k - 2ln(L)$ where $\ln(L)$ is the logarithm of the maximum likelihood from the model and k is the number of model parameters in the model. ΔAIC = AIC – AIC of the best model with the optimal α. The best model is highlighted.

Supplementary Table 27: Estimated cross-ancestry genetic correlation (SE) for Basal Metabolic Rate (BMR)

	**Cohort1/ White British (n=28,992)**	**Cohort2/ Other European (n=25,252)**	**Cohort3/ South Asian (n=5,729)**	**Cohort4/ African (n=5,775)**
**Cohort1/ White British**		**1.035 (0.036)** **P= 3.30e-01** h_c^2 (WB)= 0.293 (0.012) h_c^2 (OE)= 0.272 (0.013)	**0.904 (0.094)** **P= 3.07e-01** h_c^2 (WB)=0.269 (0.012) h_c^2 (SAS)=0.337 (0.055)	**0.790 (0.118)** **P= 7.51e-02** h_c^2 (WB)= 0.250 (0.012) h_c^2 (AFR)= 0.249 (0.053)
**Cohort2/ Other European**			**0.995 (0.100)** **P=9.60e-01** h_c^2 (OE)= 0.253 (0.013) h_c^2 (SAS)= 0.348 (0.055)	**0.449 (0.109)** **P= 4.30e-07** h_c^2 (OE)= 0.235 (0.013) h_c^2 (AFR)= 0.251 (0.055)
**Cohort3/ South Asian**				**0.752 (0.206)** **P= 2.28e-01** h_c^2 (SAS)= 0.273 (0.052) h_c^2 (AFR)= 0.251 (0.054)
**Cohort4/ African**				

P is the *p*-value based on Wald's test statistics for the null hypothesis of the estimated cross ancestry genetic correlation r_g=1 (i.e., a two-sided test). h_c^2 is the estimated SNP-based heritability from the bivariate GREML, using SNPs common between two ancestries. WB, OE, SAS, and AFR indicates White British, Other European, South Asian, and African ancestry cohorts.

Supplementary Table 28: Estimated cross- ancestry genetic correlation (SE) for whole body fat free mass

	**Cohort1/ White British (n=28,990)**	**Cohort2/ Other European (n=25,249)**	**Cohort3/ South Asian (n=5,729)**	**Cohort4/ African (n=5,774)**
**Cohort1/ White British**		**1.025 (0.034)** **P=4.62e-01** h_c^2 (WB)= 0.308 (0.012) h_c^2 (OE)= 0.295 (0.014)	**0.887 (0.088)** **P= 1.99e-01** h_c^2 (WB)=0.286 (0.012) h_c^2 (SAS)=0.339 (0.053)	**0.787 (0.114)** **P= 6.17e-02** h_c^2 (WB)= 0.265 (0.012) h_c^2 (AFR)= 0.239 (0.049)
**Cohort2/ Other European**			**1.028 (0.099)** **P=7.77e-01** h_c^2 (OE)= 0.277 (0.014) h_c^2 (SAS)= 0.274 (0.044)	**0.469 (0.107)** **P= 6.95e-07** h_c^2 (OE)= 0.258 (0.013) h_c^2 (AFR)= 0.239 (0.051)
**Cohort3/ South Asian**				**0.672 (0.197)** **P= 9.59e-02** h_c^2 (SAS)= 0.275 (0.050) h_c^2 (AFR)= 0.241 (0.050)
**Cohort4/ African**				

P is the *p*-value based on Wald's test statistics for the null hypothesis of the estimated cross ancestry genetic correlation r_g=1 (i.e., a two-sided test). h_c^2 is the estimated SNP-based heritability from the bivariate GREML, using SNPs common between two ancestries. WB, OE, SAS, and AFR indicates White British, Other European, South Asian, and African ancestry cohorts.

Supplementary Table 29: Estimated cross- ancestry genetic correlation (SE) for body fat percentage

	Cohort1/ White British (n=28,948)	Cohort2/ Other European (n=25,191)	Cohort3/ South Asian (n=5,728)	Cohort4/ African (n=5,769)
**Cohort1/ White British**		**1.102 (0.051)** **P= 4.55e-02** h_c^2 (WB)= 0.198 (0.012) h_c^2 (OE)= 0.190 (0.013)	**0.844 (0.140)** **P= 2.65e-01** h_c^2 (WB)=0.171 (0.011) h_c^2 (SAS)=0.228 (0.057)	**0.649 (0.170)** **P= 3.89e-02** h_c^2 (WB)= 0.156 (0.011) h_c^2 (AFR)= 0.186 (0.038)
**Cohort2/ Other European**			**0.966 (0.138)** **P=8.05e-01** h_c^2 (OE)= 0.179 (0.013) h_c^2 (SAS)= 0.255 (0.056)	**0.7942 (0.181)** **P= 2.55e-01** h_c^2 (OE)= 0.162 (0.012) h_c^2 (AFR)= 0.184 (0.038)
**Cohort3/ South Asian**				**1.409 (0.381)** **P= 2.83e-01** h_c^2 (SAS)=0.185 (0.051) h_c^2 (AFR)= 0.178 (0.039)
**Cohort4/ African**				

P is the *p*-value based on Wald's test statistics for the null hypothesis of the estimated cross ancestry genetic correlation r_g=1 (i.e., a two-sided test). h_c^2 is the estimated SNP-based heritability from the bivariate GREML, using SNPs common between two ancestries. WB, OE, SAS, and AFR indicates White British, Other European, South Asian, and African ancestry cohorts.

Supplementary Table 30: Estimated cross-ancestry genetic correlation (SE) for pulse rate

	Cohort1/ White British (n=27,638)	Cohort2/ Other European (n=24,156)	Cohort3/ South Asian (n=5,654)	Cohort4/ African (n=5,823)
**Cohort1/ White British**		**1.052 (0.087)** **P= 5.55e-01** $h_c^2\ (WB)$= 0.130 (0.012) $h_c^2\ (OE)$= 0.114 (0.013)	**0.899 (0.166)** **P= 5.42e-01** $h_c^2\ (WB)$=0.121 (0.011) $h_c^2\ (SAS)$= 0.176 (0.048)	**0.804 (0.259)** **P= 4.49e-01** $h_c^2\ (WB)$= 0.110 (0.011) $h_c^2\ (AFR)$= 0.115 (0.052)
**Cohort2/ Other European**			**0.909 (0.191)** **P=6.33e-01** $h_c^2\ (OE)$= 0.099 (0.012) $h_c^2\ (SAS)$= 0.162 (0.048)	**0.739 (0.265)** **P= 3.24e-01** $h_c^2\ (OE)$= 0.092 (0.011) $h_c^2\ (AFR)$= 0.119 (0.052)
**Cohort3/ South Asian**				**0.306 (0.339)** **P= 4.06e-02** $h_c^2\ (SAS)$= 0.132 (0.044) $h_c^2\ (AFR)$= 0.134 (0.053)
**Cohort4/ African**				

P is the *p*-value based on Wald's test statistics for the null hypothesis of the estimated cross ancestry genetic correlation r_g=1 (i.e., a two-sided test). h_c^2 is the estimated SNP-based heritability from the bivariate GREML, using SNPs common between two ancestries. WB, OE, SAS, and AFR indicates White British, Other European, South Asian, and African ancestry cohorts.

Supplementary Table 31: Estimated cross- ancestry genetic correlation (SE) for Education

	Cohort1/<br>White British<br>(n=29,535)	Cohort2/<br>Other European<br>(n=25,714)	Cohort3/<br>South Asian<br>(n=5,719)	Cohort4/<br>African<br>(n=5,872)
**Cohort1/<br>White British**		**0.996 (0.075)**<br>**P= 9.57e-01**<br>h_c^2 (WB)= 0.147 (0.011)<br>h_c^2 (OE)= 0.123 (0.012)	**0.517 (0.111)**<br>**P= 1.35e-05**<br>h_c^2 (WB)=0.138 (0.011)<br>h_c^2 (SAS)= 0.276 (0.053)	**0.015 (0.156)**<br>**P= 2.71e-10**<br>h_c^2 (WB)= 0.122 (0.010)<br>h_c^2 (AFR)= 0.207 (0.045)
**Cohort2/<br>Other European**			**0.495 (0.124)**<br>**P=4.64e-05**<br>h_c^2 (OE)= 0.116 (0.012)<br>h_c^2 (SAS)= 0.280 (0.053)	**0.262 (0.182)**<br>**P= 5.01e-05**<br>h_c^2 (OE)= 0.105 (0.011)<br>h_c^2 (Afr)= 0.210 (0.045)
**Cohort3/<br>South Asian**				**-0.473 (0.258)**<br>**P= 1.13e-08**<br>h_c^2 (SAS)= 0.217 (0.048)<br>h_c^2 (AFR)= 0.207 (0.046)
**Cohort4/<br>African**				

P is the *p*-value based on Wald's test statistics for the null hypothesis of the estimated cross ancestry genetic correlation r_g=1 (i.e., a two-sided test). h_c^2 is the estimated SNP-based heritability from the bivariate GREML, using SNPs common between two ancestries. WB, OE, SAS, and AFR indicates White British, Other European, South Asian, and African ancestry cohorts.

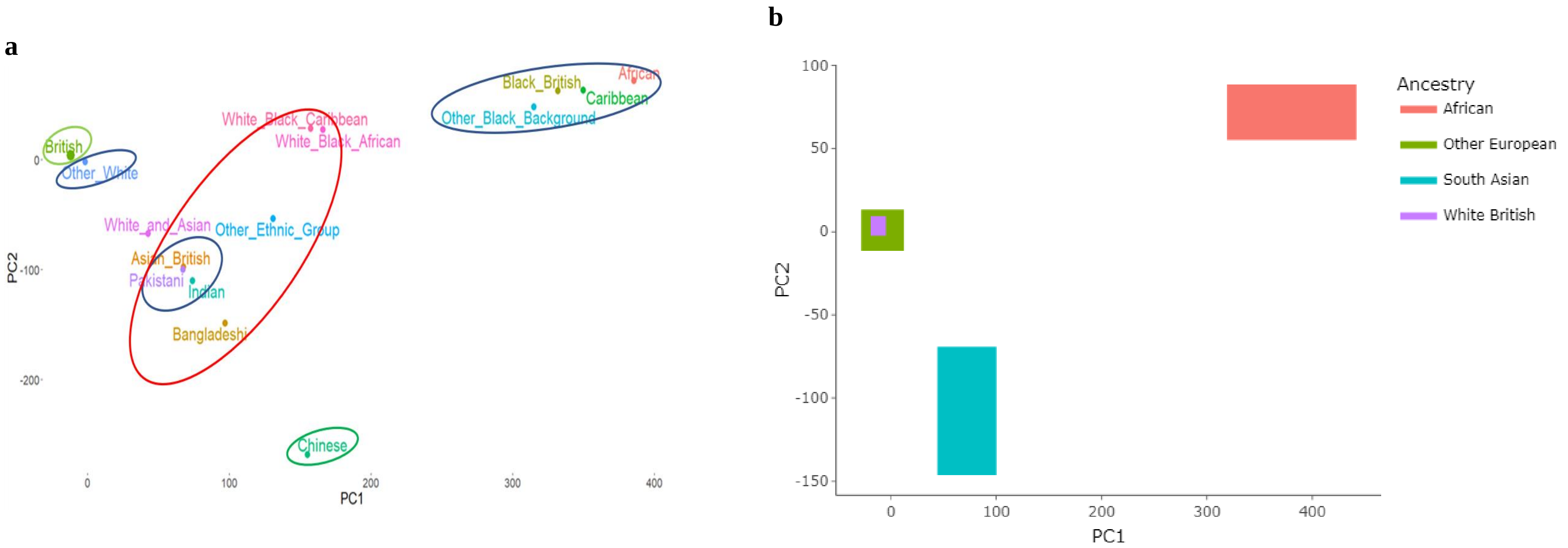

Supplementary Figure 1: Two-dimensional scatter plots using PC1 and PC2 for UK Biobank samples. a. Classification of ancestries, b. Boxes include samples within 2 standard deviations from the mean of PC1 and PC2 for the four main ancestries used in this study (about 95% of the individuals are included within the range of mean±2SD).

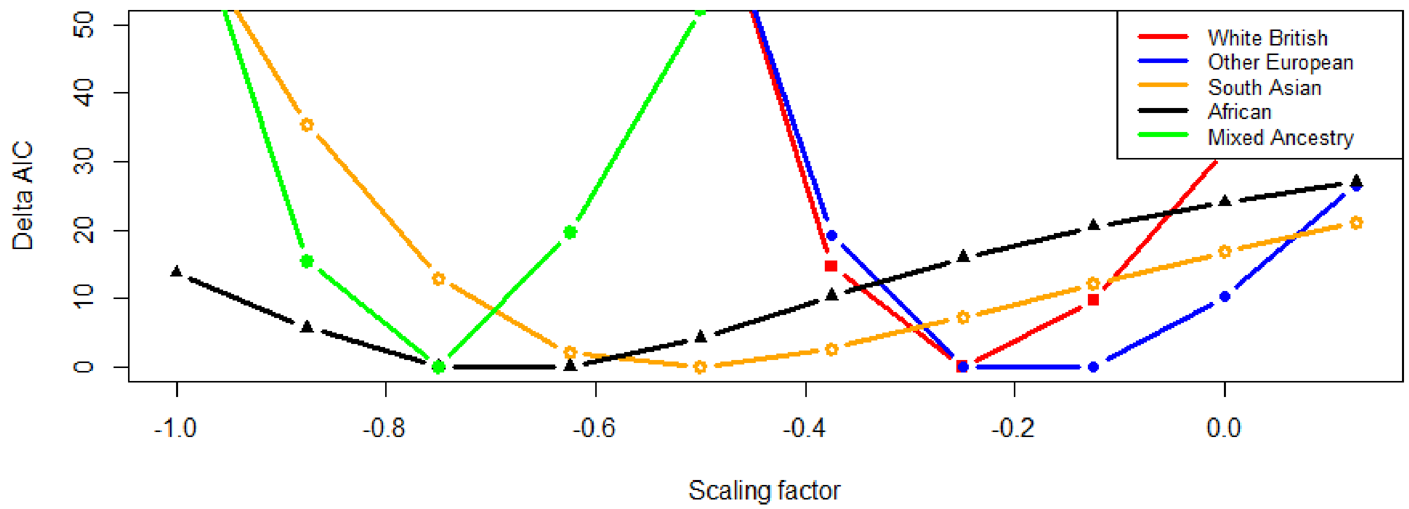

Supplementary Figure 2: Determining optimal scaling factors (α) across different ancestry groups using LDAK-thin-α model. LDAK-thin-α model assumes that SNPs contribute unequally to the heritability estimation according to their LD structure. ΔAIC values from LDAK-thin -α models are plotted against scaling factors, α, for each ancestry group. The lowest AIC (i.e. ΔAIC=0) indicates the best model. The sample sizes are 30,000, 26,457, 6,199, 6,179 and 11,797 for White British, Other European, South Asian, African, and mixed ancestry groups, respectively.

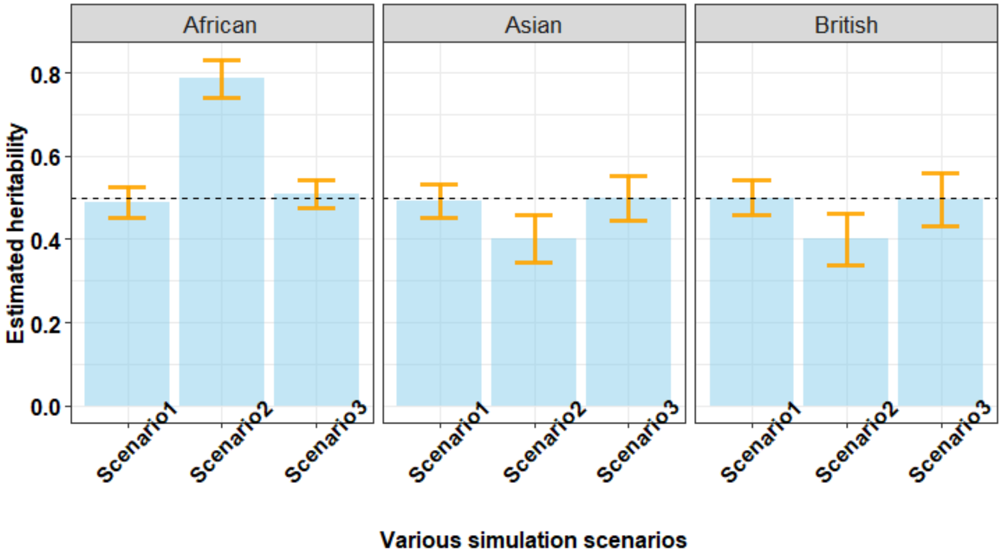

Supplementary Figure 3: Estimated heritability based on simulated phenotypes for several ancestry groups. The true heritability was 0.5 (horizontal dashed line) for simulated phenotypes using the real genotypic data after quality control in three different scenarios. Scenario 1 (simulation based α= -0.5 and GRM estimated based on α= -0.5), scenario 2 (simulation based α= -1.0 and GRM estimated based on α= -0.5), scenario 3 (simulation based α= -1.0 and GRM estimated based on α= -1.0). The main bars represent the averages of estimated heritabilities and the error bars indicate 95% confidence interval (CI) of the averages, which are derived from 500 experimental replicates that were independently carried out. For simulation, the total number of individuals was 1000 and the total number of SNPs was 500,000 in each ancestry (White British, South Asian and African ancestry cohorts). Simulation was based on 1000 random common SNPs as causal. The causal effect of 1000 random SNPs was estimated following a normal distribution with mean 0 for the phenotypes within ancestry group and causal effects of each SNPs were scaled by reference allele frequencies. All simulation process was implemented in MTG2. GRM for scenario1 and scenario 2 were implemented in PLINK[2] and GRM for scenario3 was estimated in LDAK software[3].

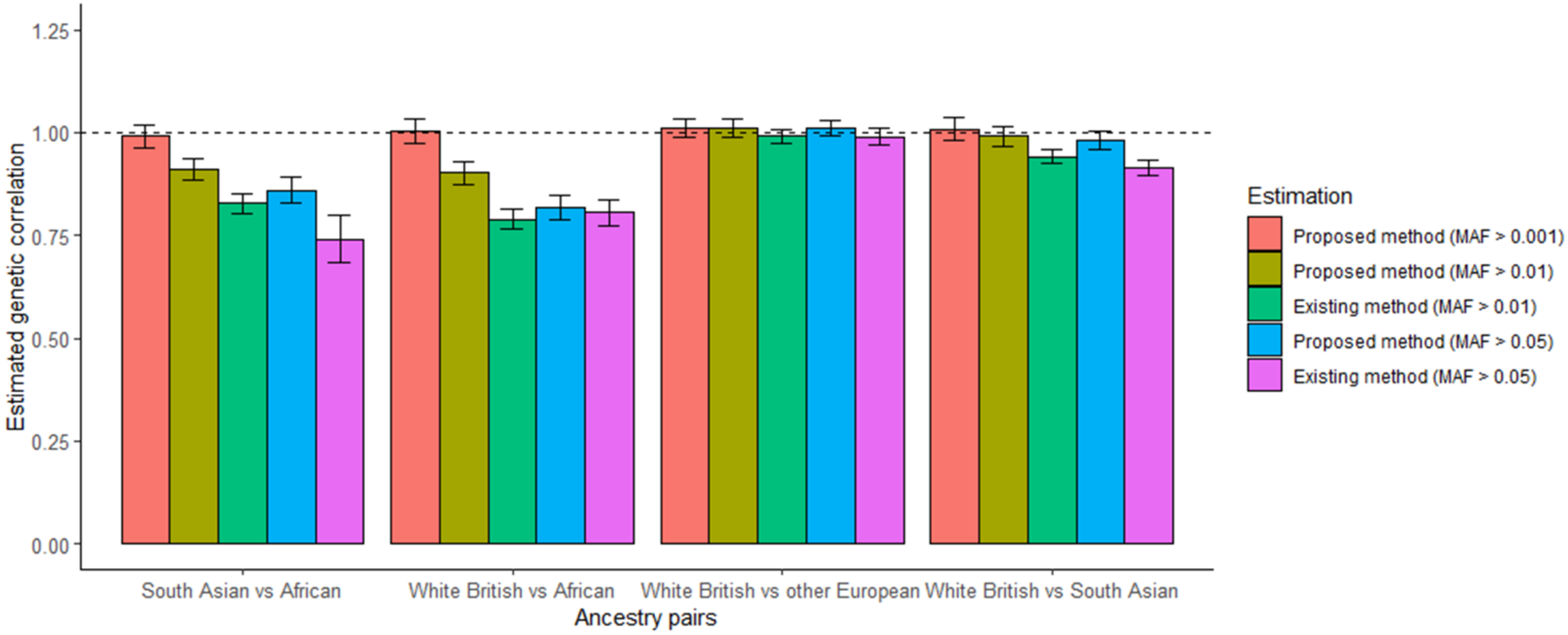

Supplementary Figure 4: Estimated cross-ancestry genetic correlations can be biased when the causal SNPs are not 100% common between ancestries, and the biasedness can be reduced by using the proposed method. The main bars are the averages of estimated cross-ancestry genetic correlations and the error bars indicate 95% confidence interval (CI) of the averages, which are derived from 100 experimental replicates that were independently carried out. The phenotypic simulation was based on the real genotypes, using ancestry-specific alphas (estimated from the real data as shown in Figure 1). The sample size used in this simulation was 5,000 for each ancestry (total 10,000). After QC including MAF < 0.001, the number of SNPs was ~200,000 across ancestry pairs, among which 10,000 SNPs were selected as causal. The true cross-ancestry genetic correlation was simulated as 1. For the genotypic data, QC of MAF < 0.01 or MAF < 0.05 was applied for each ancestry to generate a situation that causal SNPs are not 100% common between ancestries. In this situation, the proposed and existing methods were applied.

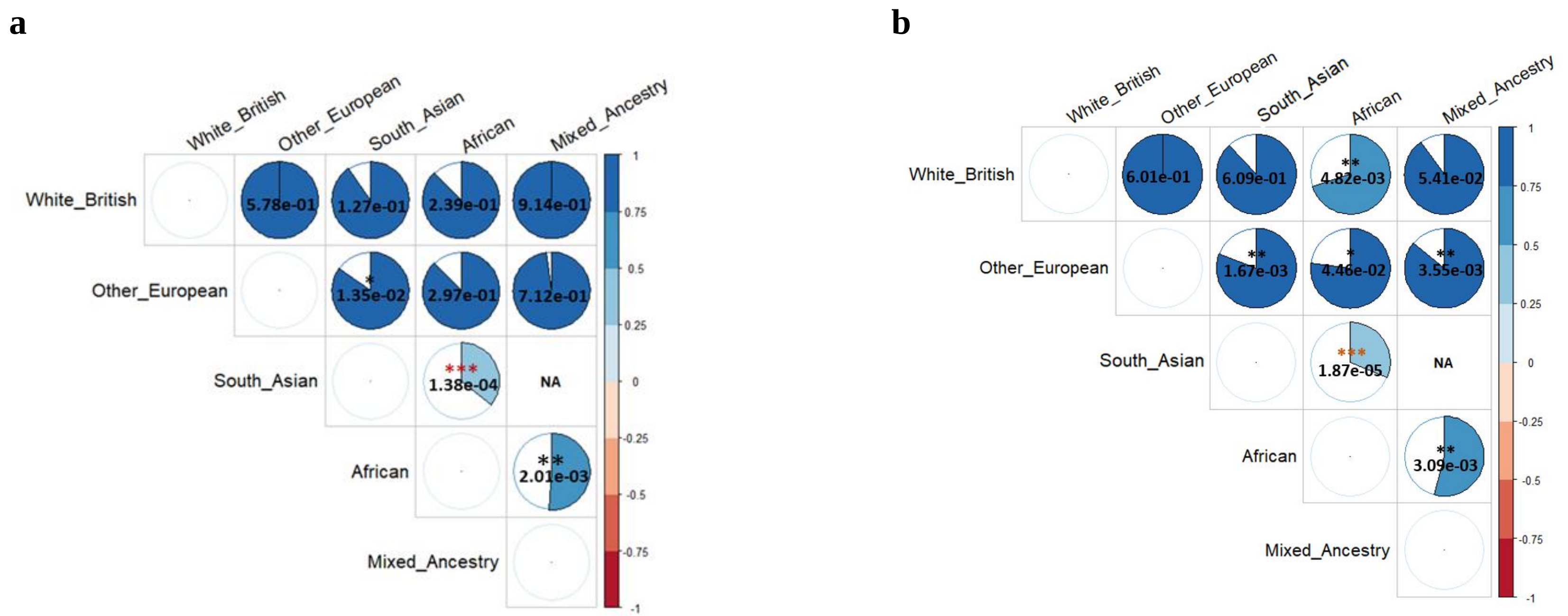

Supplementary Figure 5: Comparison of the estimations of cross-ancestry genetic correlations of standing height between proposed and existing methods. a. Estimated cross-ancestry genetic correlations of standing height using proposed method and b. Estimated cross-ancestry genetic correlations of standing height using existing method. The colour and size of each pie chart indicates the magnitude of estimated cross-ancestry genetic correlations. The value in each pie chart is a *p*-value (*, **, *** indicates p value < 0.05, < 0.01 and <0.001, respectively) based on Wald's test statistics for testing the null hypothesis of r_g=1 (i.e., a two-sided test). Over-interpretation (African and European; Other European and mixed ancestry) in existing method is due inaccurate consideration of α. Coloured asterisk indicates significantly different from 1 after Bonferroni correction (0.05/54).

a **b**

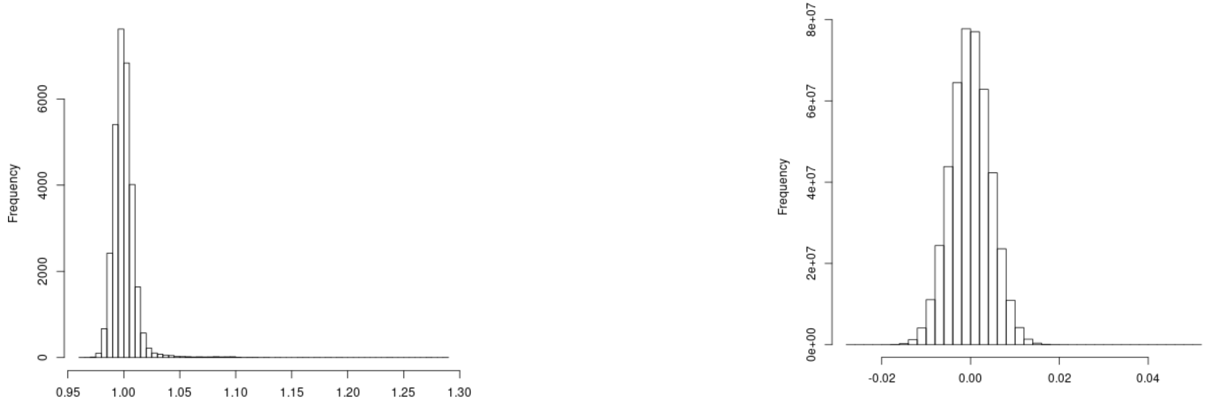

Supplementary Figure 6: Distribution of diagonal values (a) and off diagonal values (b) of GRM (GCTA model with α = -0.5) in White British ancestry cohort. Means of diagonal and off diagonal of elements are 1.0002 and -3.33e-05, respectively.

a **b**

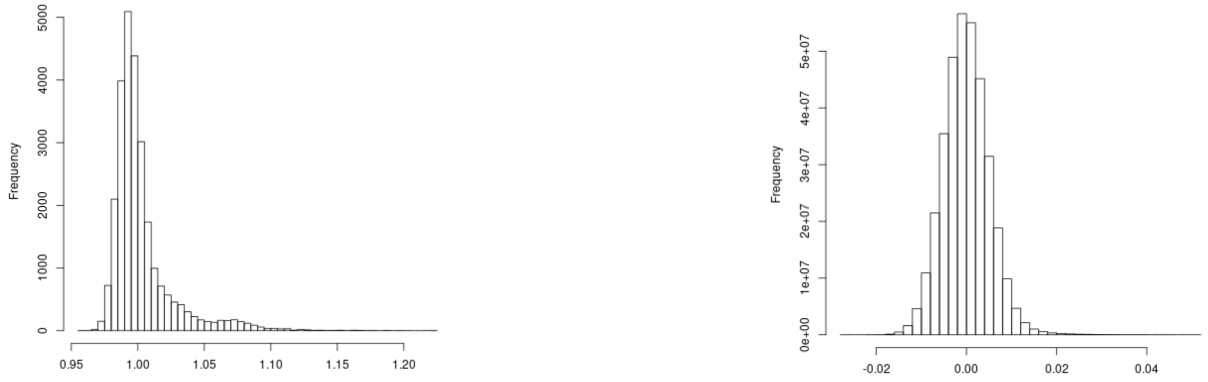

Supplementary Figure 7: Distribution of diagonal values (a) and off diagonal values (b) of GRM (GCTA model with α = -0.5) in Other European ancestry cohort. Means of diagonal and off diagonal of elements are 1.0004 and -2.23e-05, respectively.

a

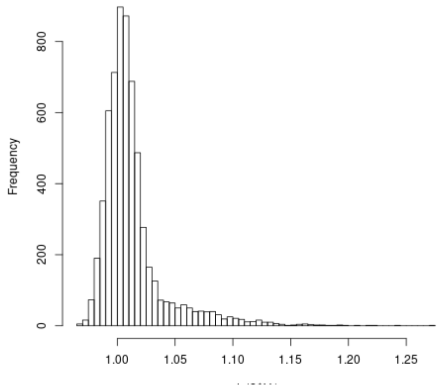

b

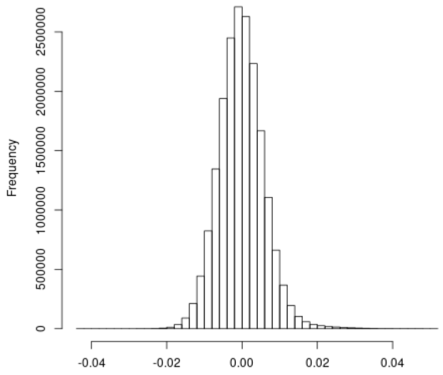

Supplementary Figure 8: Distribution of diagonal values (a) and off diagonal values (b) of GRM (GCTA model with α = -0.5) in South Asian ancestry cohort. Means of diagonal and off diagonal of elements are 1.0128 and -1.61e-04, respectively.

a **b**

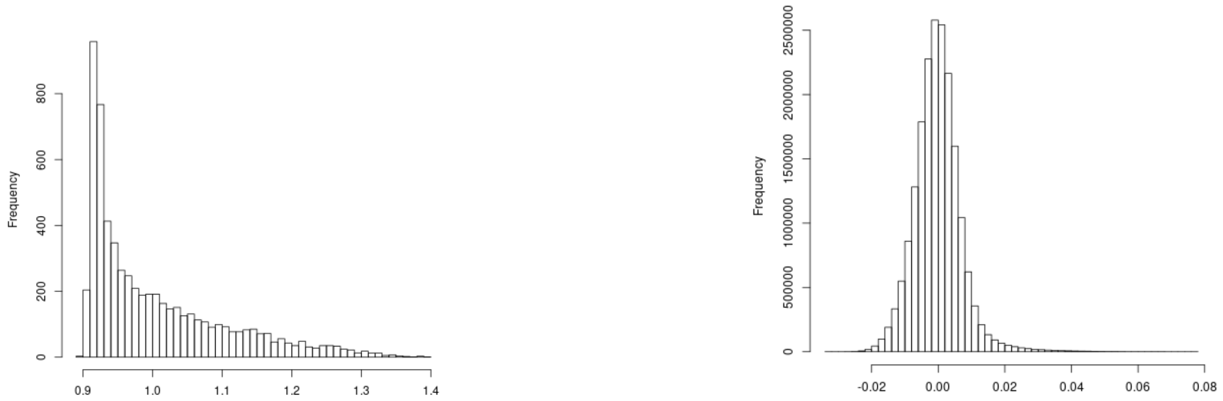

Supplementary Figure 9 Distribution of diagonal values (a) and off diagonal values (b) of GRM (GCTA model with α = -0.5) in African ancestry cohort. Means of diagonal and off diagonal of elements are 1.0033 and -1.65e-04, respectively**.**

a b

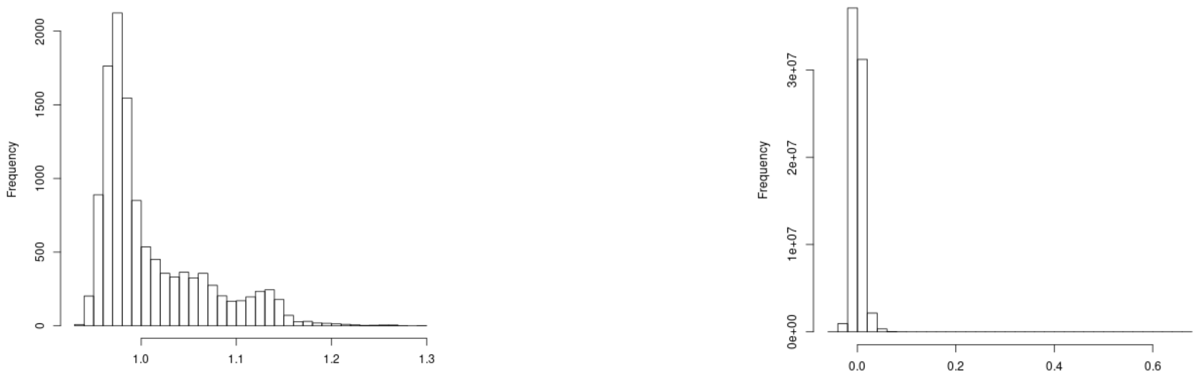

Supplementary Figure 10: Distribution of diagonal values (a) and off diagonal values (b) of GRM (GCTA model with α = -0.5) in mixed ancestry cohort. Means of diagonal and off diagonal of elements are 1.0096 and -8.57e-04, respectively.

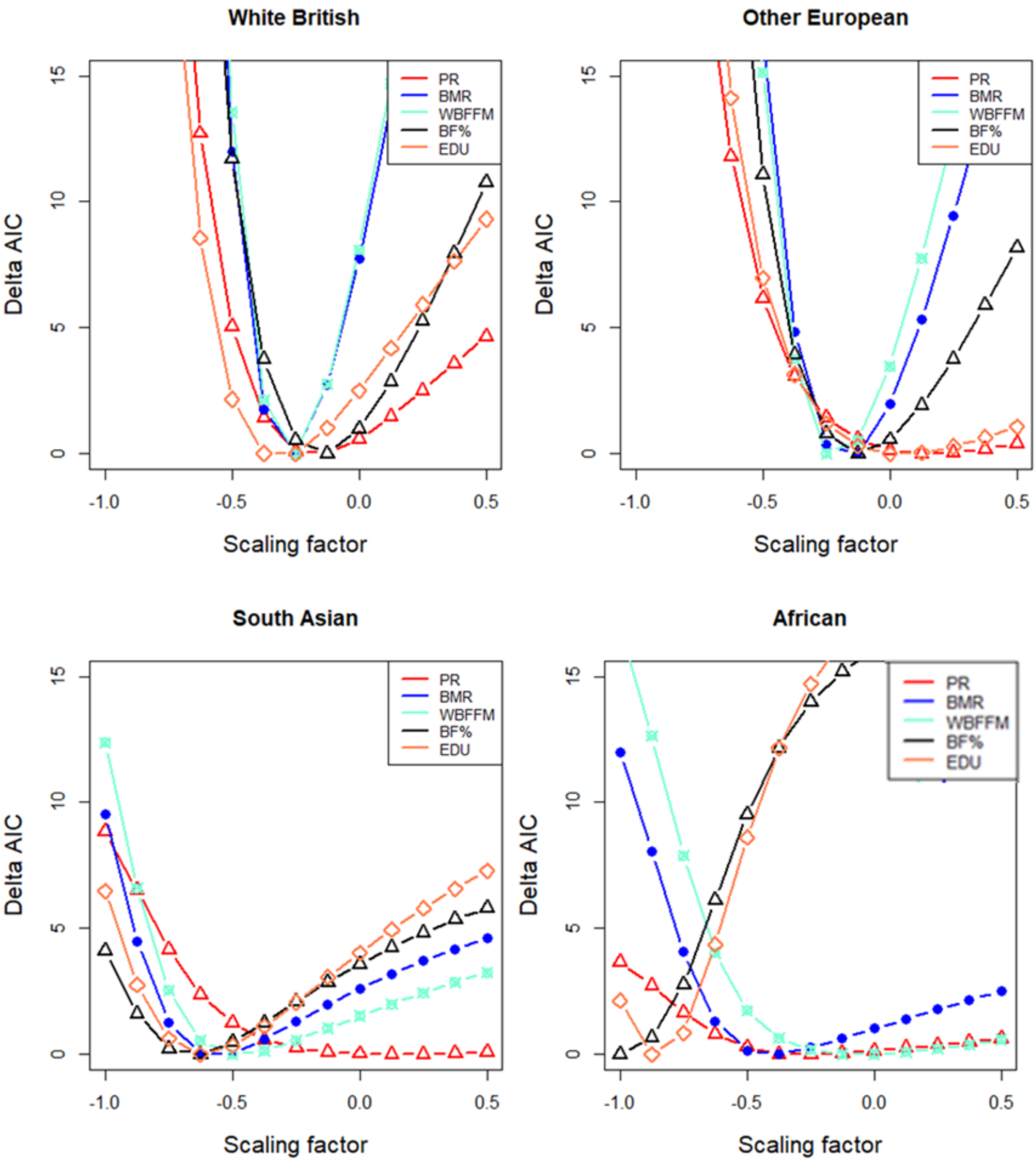

Supplementary 11: Determining optimal scaling factors for a broader range of complex traits across ancestries using GCTA-α models. GCTA-α model assumes that all SNPs have an equal contribution to the heritability estimation and α value varies across ancestries[3,4]. ΔAIC values are plotted against scaling factors, α, for each ancestry group for each trait. The lowest AIC (i.e., ΔAIC=0) indicates the best model. The sample sizes are 30,000, 26,457, 6,199, and 6,179 for White British, Other European, South Asian, and African ancestry groups, respectively. Trait labels are: PR= pulse rate, BMR= basal metabolic rate, WBFFM= whole Body fat free mass, BF%= body fat percentage, EDU= educational attainment.

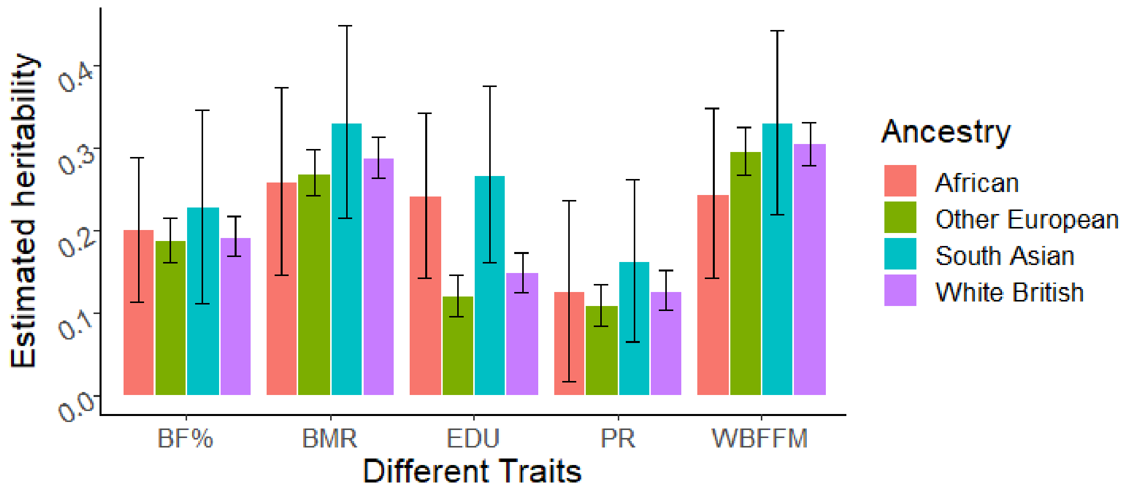

Supplementary Figure 12: Estimated SNP-based heritability across ancestries for a broad range of complex traits. The main bars indicate estimated SNP-based heritability based on trait and ancestry specific scale factor and the error bars indicate 95% confidence intervals (CI). PR= pulse rate, BMR= basal metabolic rate, WBFFM= whole body fat free mass, BF%= body fat percentage, EDU= educational attainment. The total sample sizes were 30000, 26457, 6199, and 6179 for White British, Other European, South Asian, and African ancestry cohorts.

Supplementary Notes

Cross ancestry genetic correlation using Popcorn

We have estimated LD score for each pair of ancestry as recommended by the author. The basic command to compute LD scores for two populations:

```
popcorn compute -v 1 --bfile1 ancestry1 --bfile2 ancestry2 scores.txt
```

Pre-processing of data summary statistics is not supported by Popcorn. GWAS summary statistics for each ancestry group was separately estimated by PLINK2, following the Popcorn manual. Using the summary statistics and LD scores, we used the following Popcorn command to estimate cross ancestry genetic correlations.

The command to compute the heritability and genetic correlation:

```
popcorn fit -v 1 --cfile scores.txt --gen_effect --sfile1
ancestry1_sumstats.txt --sfile2 ancestry2_sumstats.txt
correlation_output.txt
```

This is an ideal case for popcorn when individual level genotypes are available. We also tested using LD scores provided by popcorn authors, and found the estimates were more biased (result not shown).

Cross ancestry genetic correlation using XPASS

XPASS was used by following the manual (https://github.com/YangLabHKUST/XPASS/blob/master/Manual_XPASS.pdf). The command to estimate genetic correlations

```
library(devtools)
library(XPASS)
library(data.table)
# Reference genotypes for ancestry1 (prefix of plink file bim/bed/fam)
ref_1 <- "ancestry1"
# Reference genotypes for ancestry2 (prefix of plink file bim/bed/fam)
ref_2 <- "ancestry2"
# sumstats of height
summary1 <- "ancestry1.summary_xapass" # target
summary2 <- "ancestry2.summary_xapass" # auxiliary
# Covariates
cov_1="ancestry1_cov"
cov_2="ancestry2_cov"

correl_out <-XPASS(file_z1 = summary1,
                file_z2 = summary2,
                file_ref1 = ref_1,
                file_ref2 = ref_2,
                file_cov1 = cov_1,
                file_cov2 = cov_2,
                compPRS=T,
                sd_method="LD_block",compPosMean = T,
                file_out = "XPASS_out")

out=correl_out$H
```

Supplementary References

1. Lee, S.H. & Van der Werf, J.H. MTG2: an efficient algorithm for multivariate linear mixed model analysis based on genomic information. *Bioinformatics* **32**, 1420-1422 (2016).
2. Purcell, S. *et al.* PLINK: a tool set for whole-genome association and population-based linkage analyses. *The American Journal of Human Genetics* **81**, 559-575 (2007).
3. Speed, D., Hemani, G., Johnson, M.R. & Balding, D.J. Improved heritability estimation from genome-wide SNPs. *The American Journal of Human Genetics* **91**, 1011-1021 (2012).
4. Speed, D., Cai, N., Johnson, M.R., Nejentsev, S. & Balding, D.J. Reevaluation of SNP heritability in complex human traits. *Nature Genetics* **49**, 986-992 (2017).
