# Supplementary material for: A method for an unbiased estimate of cross-ancestry genetic correlation using individual-level data
Source: Nat Commun. 2023 Feb 9;14:722. doi: 10.1038/s41467-023-36281-x (PMC9911789; doi:10.1038/s41467-023-36281-x)
Supplement: Supplementary file 3 — Reporting Summary [file 41467_2023_36281_MOESM3_ESM.pdf]

nature portfolio

Corresponding author(s): Sang Hong Lee

Last updated by author(s): Jan 18, 2023

Reporting Summary

Nature Portfolio wishes to improve the reproducibility of the work that we publish. This form provides structure for consistency and transparency in reporting. For further information on Nature Portfolio policies, see our Editorial Policies and the Editorial Policy Checklist.

Statistics

For all statistical analyses, confirm that the following items are present in the figure legend, table legend, main text, or Methods section.

n/a	Confirmed	
☐	☒	The exact sample size (*n*) for each experimental group/condition, given as a discrete number and unit of measurement
☐	☒	A statement on whether measurements were taken from distinct samples or whether the same sample was measured repeatedly
☐	☒	The statistical test(s) used AND whether they are one- or two-sided<br>*Only common tests should be described solely by name; describe more complex techniques in the Methods section.*
☐	☒	A description of all covariates tested
☐	☒	A description of any assumptions or corrections, such as tests of normality and adjustment for multiple comparisons
☐	☒	A full description of the statistical parameters including central tendency (e.g. means) or other basic estimates (e.g. regression coefficient) AND variation (e.g. standard deviation) or associated estimates of uncertainty (e.g. confidence intervals)
☐	☒	For null hypothesis testing, the test statistic (e.g. *F*, *t*, *r*) with confidence intervals, effect sizes, degrees of freedom and *P* value noted<br>*Give P values as exact values whenever suitable.*
☒	☐	For Bayesian analysis, information on the choice of priors and Markov chain Monte Carlo settings
☒	☐	For hierarchical and complex designs, identification of the appropriate level for tests and full reporting of outcomes
☐	☒	Estimates of effect sizes (e.g. Cohen's *d*, Pearson's *r*), indicating how they were calculated

Our web collection on statistics for biologists contains articles on many of the points above.

Software and code

Policy information about availability of computer code

Data collection	All data used for analysis in this publication were derived from the data available in the UK Biobank, described and explorable within their online Data Showcase (https://biobank.ndph.ox.ac.uk/showcase/).Data collection was done centrally by the UK Biobank. We did not use any software for data collection.
Data analysis	We used MTG2 [v2.22], GCTA[v1.93], PLINK[v2], LDAK [v5.1], Popcorn [v1.0], XPASS [v0.1.0], R[v4.3]. Source code of mtg2 can be accessed from https://github.com/mommy003/XA_GRM. Simulated phenotypic data can be reproduced using R script available in https://github.com/mommy003/XA_GRM.

For manuscripts utilizing custom algorithms or software that are central to the research but not yet described in published literature, software must be made available to editors and reviewers. We strongly encourage code deposition in a community repository (e.g. GitHub). See the Nature Portfolio guidelines for submitting code & software for further information.

Data

Policy information about availability of data

All manuscripts must include a data availability statement. This statement should provide the following information, where applicable:

- Accession codes, unique identifiers, or web links for publicly available datasets
- A description of any restrictions on data availability
- For clinical datasets or third party data, please ensure that the statement adheres to our policy

The UK Biobank data can be accessed through procedures described in its web-page (https://www.ukbiobank.ac.uk/). Simulated phenotypic data can be reproduced using R script available in https://github.com/mommy003/XA_GRM

Human research participants

Policy information about studies involving human research participants and Sex and Gender in Research.

Reporting on sex and gender	Male/female information is based on self-reporting. Sex and gender were not considered in study design, and sex- and gender-based analyses were not performed in our study. It is not likely that our findings apply to only one sex or gender
Population characteristics	The UK Biobank data consist of 500,000 participants aged between 40-69 years between 2006-1010, with ~54% females and ~46% males. Initially 50,000 participants were genotyped by Affymetrix UK BiLEVE Axiom array and remaining 450,000 genotyped by Affymetrix UK Biobank Axiom® array. Genotypes were imputed to the whole genome level with IMPUTE3 using UK10K and 1000 Genome Phase 3 as the reference.
Recruitment	The recruitment includes a half million of people from all around of UK who are currently aged 40-69 because of the age group involves people at the risk over the next few decades of developing a wide range of important diseases, conditions, and covariates. The phenotypic and genotypic information is based on extensive baseline questionnaire and physical measures, as well as stored blood and urine samples that allow many different types of assay , incorporated with information from the UK National Health Service. The UK Biobank may not be the representative of the general population due to low response rate and the "healthy Volunteer" selection bias (PMID 28641372). However, the effects of bias appear to be subtle in a recent study (PMID 26849114). The risks of participants suffering harm as a result of taking part are minimal, and UK Biobank has insurance in place to provide compensation for any negligent harm caused by participation
Ethics oversight	The UK Biobank scientific protocol of which has been reviewed and approved by the North West Multi-center Research Ethics Committee, National Information Governance Board for Health & Social Care (NIGB), and Community Health Index Advisory Group. UK Biobank has obtained informed consent from all participants. Our access to the UK Biobank data was under the reference number 14575. The research ethics approval of this study has been obtained from the University of South Australia Human Research Ethics Committee.

Note that full information on the approval of the study protocol must also be provided in the manuscript.

Field-specific reporting

Please select the one below that is the best fit for your research. If you are not sure, read the appropriate sections before making your selection.

☒ Life sciences ☐ Behavioural & social sciences ☐ Ecological, evolutionary & environmental sciences

For a reference copy of the document with all sections, see nature.com/documents/nr-reporting-summary-flat.pdf

Life sciences study design

All studies must disclose on these points even when the disclosure is negative.

Sample size	After quality control, the cleaned data includes 30000, 26457, 6199, 6179 and 11797 participants, and the total number of SNP was 1154490, 1148504, 939512, 729534 and 513362 for white British, other European, Asian, African, and mixed ancestry cohorts. Participants of the UK Biobank were stratified into multiple ancestries according to their underlying genetic ancestry based on a principal component analysis. We calculated power of our analysis using GCTA-GREML power calculator (https://shiny.cnsgenomics.com/gctaPower/).<br><br>For simulation we have used random 1000 individuals and 500000 SNPs from each of the ancestry population for estimating cross-ancestry genetic correlation. For real data analysis of cross-ancestry genetic correlation for complex traits (BMI, Standing height, Waist circumference, Hip, circumference, Waist-Hip ratio, weight, Basal metabolic rate, Body fat percentage, Whole body fat free mass, Pulse rate and Education) across ancestries.
Data exclusions	We applied well-established (hence widely used) quality control (QC) procedures to exclude unreliable genetic data. The QC was performed for each of the ancestry population separately. The data exclusion was performed before simulation and main analysis. The rationale behind the data exclusion is to avoid bias in our results due to poor quality of data. The QC control procedure is described as follows. The QC criteria

	include an INFO score (an imputation reliability) ≥0.6, SNP missingness <0.05, minor allele frequency (MAF) >0.01, Hardy–Weinberg equilibrium p-value > 10-04. We also excluded individuals outside ±6 SD of the population mean for first and second ancestry principal components. Individuals with genetic relatedness ≥0.05 were excluded from each ancestry group. In the analysis, we retained HapMap3 SNPs only as these are high in quality and well calibrated to dissect genetic architecture of complex traits
Replication	For comparing existing methods and proposed methods, we have used 500 replication during simulation. It is confirmed by simulation that the proposed method outperformed existing methods.
Randomization	This study does not involved any experimental groups. So description of samples allocation into experimental groups are not applicable
Blinding	Blinding is not applicable to our study, because the data were collected by the UK Biobank and the ID of the participants has been encrypted

Reporting for specific materials, systems and methods

We require information from authors about some types of materials, experimental systems and methods used in many studies. Here, indicate whether each material, system or method listed is relevant to your study. If you are not sure if a list item applies to your research, read the appropriate section before selecting a response.

Materials & experimental systems

n/a	Involved in the study
☒	☐ Antibodies
☒	☐ Eukaryotic cell lines
☒	☐ Palaeontology and archaeology
☒	☐ Animals and other organisms
☒	☐ Clinical data
☒	☐ Dual use research of concern

Methods

n/a	Involved in the study
☒	☐ ChIP-seq
☒	☐ Flow cytometry
☒	☐ MRI-based neuroimaging
